# Who venerated the ancestors at the Petit-Chasseur site? Examining Early Bronze Age cultic activities around megalithic monuments through the archaeometric analyses of ceramic findings (Upper Rhône Valley, Switzerland, 2200–1600 BC)

**DOI:** 10.1007/s12520-023-01737-0

**Published:** 2023-04-20

**Authors:** Delia Carloni, Branimir Šegvić, Mario Sartori, Giovanni Zanoni, Marie Besse

**Affiliations:** 1 Department F.-A. Forel for Environmental and Aquatic Sciences, Laboratory of Prehistoric Archaeology and Anthropology, University of Geneva, Geneva, Switzerland; 2 Department of Geosciences, Texas Tech University, Lubbock, TX USA; 3 Department of Earth Sciences, University of Geneva, Geneva, Switzerland

**Keywords:** Pottery, Early Bronze Age, Ceramic traditions, Human–environment relationship, Ancestor cult, Social ties

## Abstract

Through the analyses of recovered pottery, this study explores the social dimension of an ancestor cult developed at the Petit-Chasseur megalithic necropolis (Upper Rhône Valley, Switzerland) during the Early Bronze Age (2200–1600 BC). The jar votive offerings and domestic pottery from settlement sites were characterized using a range of spectroscopic and microscopic techniques. Acquired archaeometric data allowed identification of six ceramic fabrics and two types of clay substrate—illite- and muscovite-based—which were used in pottery production. The present article discusses the pottery composition in the light of natural resources available in the region, thus shedding light on raw material choices and paste preparation recipes. The Early Bronze Age people that lived in the Upper Rhône Valley seem to have shared a common ceramic tradition, partly inherited from the previous Bell Beaker populations. The compositional correspondence between the jar offerings and domestic pottery revealed that the majority of the known Early Bronze Age groups partook in cultic activities at the Petit-Chasseur megalithic necropolis.

**Supplementary Information:**

The online version contains supplementary material available at 10.1007/s12520-023-01737-0.

## Introduction

The megalithic monuments of the Petit-Chasseur site^1^ are known for their importance for the Final Neolithic (FN) (3100–2450 BC) and Bell Beaker (BB) (2450–2200 BC) communities of the Upper Rhône Valley (URV) in Western Switzerland (Gallay 1995; Harrison and Heyd 2007; Besse et al. 2011, 2014). During the 3rd millennium BC, the site was used to ostentatiously display wealth and play out social competition, thus acting as a symbolic landscape within both the living and the dead had their place (Gallay 1978; Gallay 1995, 2007, 2014, 2016; Parker Pearson 1993; Testart 2005, 2014; Corboud and Curdy 2009). At the turn of the 2nd millennium BC, the necropolis continued to play a great role in the social life of the URV communities (Gallay 1995). During the Early Bronze Age (EBA) (2200–1600 BC), the BB and FN monuments became the objects of an ancestor cult, consisting of ritual depositions of jars, offerings of faunal remains, and construction of cairns (Bocksberger 1976, 1978; Gallay and Chaix 1984; Gallay 1989, 1995). Stratigraphy and radiocarbon dates point out that veneration activities at the Petit-Chasseur site lasted for almost the entire EBA, which is four or five centuries at least (Gallay and Chaix 1984; Besse et al. 2011; Gallay 2016; Derenne et al. 2020). The ancestor cult evolved around the FN and BB monumental tombs of the Petit-Chasseur necropolis testifies to the high level of engagement the EBA communities of the URV had developed with this place (Gallay and Chaix 1984; Gallay 1995, 2016; Harrison and Heyd 2007). Megalithic monuments usually commemorate individuals as well as the events in which they were involved and act as permanent memorials, capable of passing on meanings and messages between generations (e.g. Parker Pearson 1993; Parker Pearson and Ramilisonina 1998; Scarre and Insoll 2011, and references therein). At the same time, the dead were generally believed to have had power over a variety of aspects of the livings’ everyday life, such as agricultural fertility, maintenance of the social order, embodiments of land rights, and conservation of traditions (Parker Pearson 1993).

Ancestor cults must be analyzed in light of the link between the world of the dead and that of the living by adopting a contextual approach (Parker Pearson 1993; Parker Pearson and Ramilisonina 1998; Insoll 2004, 2009, 2011; Renfrew 2007). The relationship between the living and the dead can be reconstructed by examining the veneration-related material remains (Parker Pearson 1993; Insoll 2011; Scarre and Insoll 2011). In the case of the Petit-Chasseur necropolis, previous analyses of ceramic findings provided a range of information. The typological classification showed that all fragmented vessels belonged to large storage containers which were all made following a common stylistic and technical tradition (Gallay and Chaix 1984; Carloni et al. 2020, 2021; Derenne et al. 2020). However, the archaeometric study revealed that these containers were manufactured using different types of raw materials and following a diversity of co-existing paste preparation recipes (Carloni et al. 2021). The selection of the raw material was likely influenced by the type of natural resources available at different altitude zones of the URV (Carloni et al. 2021). Cited researchers therefore inferred that the ancestor cult was practiced by a variety of groups which dwelled in the distinct areas of the URV and gathered around megalithic monuments to build and maintain social ties (Gallay and Chaix 1984; Gallay 1995, Gallay 2016; Moinat and Gallay 1998). The research presented in this paper aims to test such inferences by comparing EBA jars of the Petit-Chasseur necropolis with domestic pottery of contemporaneous settlement sites from a perspective of raw material choices and paste preparation recipes. The fabrication process of ceramics is an interplay between functional, environmental, and cultural factors, which may result in uniformity in technical traditions or lack of it among social groups occupying the same region (Arnold 1985; Rice 1987; Orton et al. 1993; Lechtman 1994; Stark 1998; Velde and Druc 1998; Costin 2000; Skibo and Schiffer 2008; Roux 2010, 2019; Shennan 2013; Michelaki et al. 2015). Similar raw material choices and paste preparation recipes documented in both jar offerings and domestic pottery may, in this case, represent an indirect evidence of which URV communities actively participated in the rituals that took place around the megalithic monuments.

An in-depth investigation of ceramic containers by means of multiple microscopic and spectroscopic techniques led to a thorough material characterization of the pottery. Acquired data was then used (1) to unveil the raw material choices, (2) to reconstruct the paste preparation recipes followed for manufacturing the EBA domestic pottery, and (3) to compare technical traditions attested at the megalithic cemetery with those found at settlement sites. By examining the EBA cultic activities around megalithic monuments through the analyses of ceramic findings, this work explores the social dimension of monumental burials and contributes to broader research on the symbolic and sacred sphere of the European EBA societies (e.g., Kristiansen 2011, 2013; Brück and Fontijn 2013; Heyd 2013; Besse and Giligny 2021; Soares Lopes and Gomes 2021).

## Archaeological background

### The Petit-Chasseur megalithic necropolis during the Early Bronze Age

**Fig. 1:**
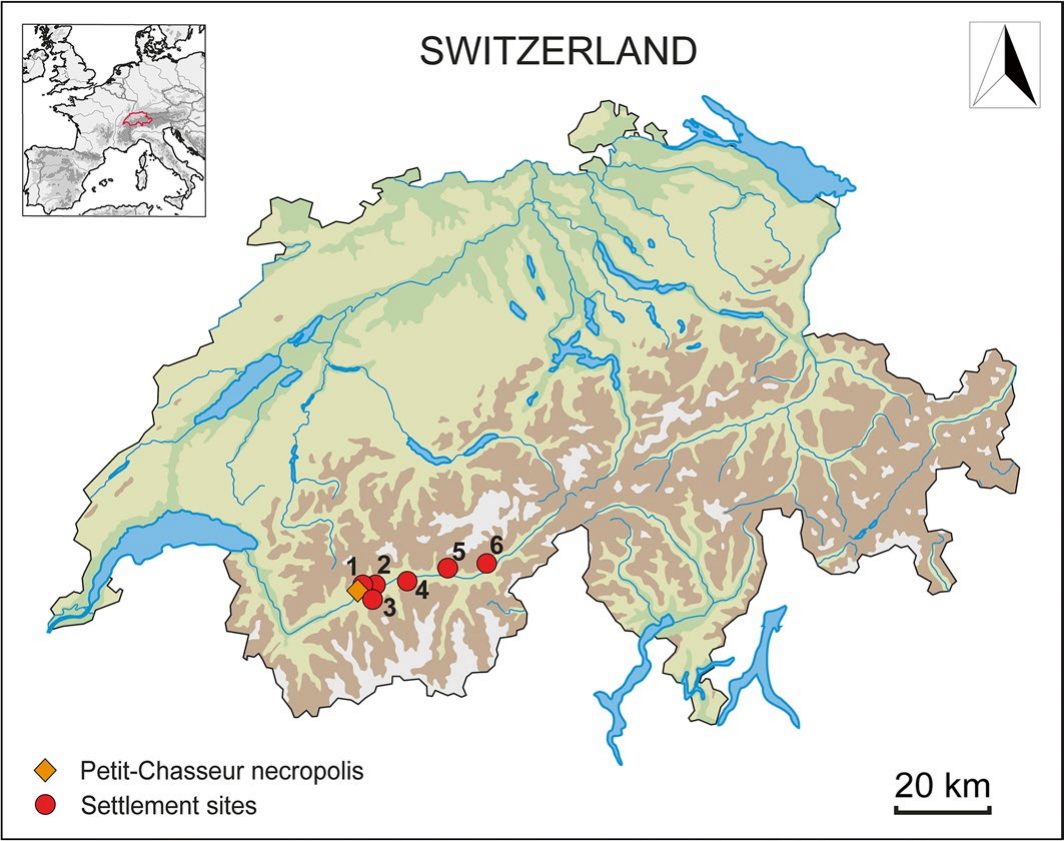
Location of the Petit-Chasseur necropolis within Switzerland and relative position of the Early Bronze Age sites selected for the study: (1) Sion *‘Petit-Chasseur III’*; (2) Sion *‘Sous-le-Scex’*; (3) Vex *‘Le Château’*; (4) Salgesch *‘Mörderstein’*; (5) Rarogne *‘Heidnischbühl II’*; (6) Naters *‘Altersheim’*. Adapted from data provided by the Swiss Federal Office of Topography (https://map.geo.admin.ch/)

**Fig. 2:**
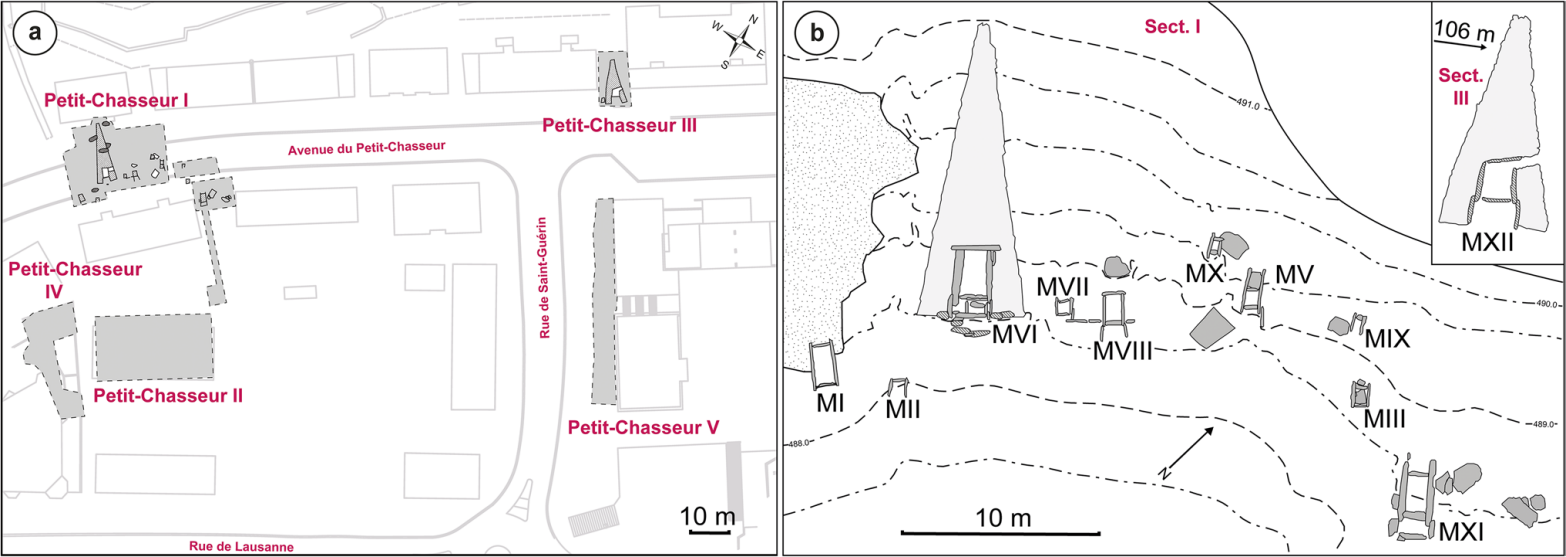
Topography of the Petit-Chasseur site: **a** excavated sectors (adapted from Mariéthoz 2009) and **b** plan of the necropolis with location of the dolmens (MI, MV, MVI, MXI, and MXII) and the cists (MII, MIII, MVII, MVIII, MIX, MX, MXIII) (adapted from Corboud and Curdy 2009)

The Petit-Chasseur site is located at the heart of the URV, in the modern city of Sion, Switzerland (Fig. 1). The 3rd millennium BC megalithic necropolis was discovered in the excavation areas *‘Petit-Chasseur I’* and *‘Petit-Chasseur III’* (Fig. 2a) (Mariéthoz 2009; Corboud and Curdy 2009; Besse et al. 2011). Monumental tombs were made using lithic slabs and are of two types: (1) dolmens having an entrance on the eastern side and (2) cists lacking an entrance (Besse et al. 2011). Among the total 12 megalithic burials discovered at the site (Bocksberger 1976, 1978; Gallay and Chaix 1984; Gallay 1989), only nine from the *‘Petit-Chasseur I’* area continued to be used during the EBA (2200–1600 BC) (Fig. 2b). MXII and MXIII situated in the *‘Petit-Chasseur III’* sector were already buried by sediments and no longer visible at that time (Favre and Mottet 2011). Based on stratigraphic information, the excavators recognized four phases named EBA I, II, III, and IV (Gallay and Chaix 1984). The complex sequence of events occurring in the cemetery during the EBA is detailed in Table 1, which was compiled based on information extracted from the works of Bocksberger (1976, 1978), Gallay and Chaix (1984), and Gallay (1989). This section contains only general observations which can be made in regard to the history of the megalithic tombs located in the sector *‘Petit-Chasseur I’* during the EBA.

**Table 1:** Detailed sequence of events occurring at Sion *‘Petit-Chasseur I’* megalithic necropolis. The first column presents activities conducted around MXI, the key context for analyzing and interpreting the Early Bronze Age (EBA) use of the Final Neolithic (FN) and Bell Beaker (BB) monumental burials (Gallay and Chaix 1984). The following columns outline contemporaneous activities conducted in the other monuments, presented according to their respective distance from MXI, from east to west (see Fig. 2b). The early sequence of events for MIX, MV, MX, MVII, MVIII, and MI cannot unequivocally assigned to the Early Bronze Age I or II

	MXI	MIII	MIX	MV, MX	MVII, MVIII	MVI	MII	MI
EBA I	• Perturbation of BB burials and relocation of some bell-shaped beakers outside the chamber, original entrance of the dolmen permanently sealed	• Violation of BB burials and relocation of the human remains outside the chamber, next to the northern slab				/	/	
	• Two ‘adventitious cists’ built on the southern and western side of the dolmen A three to four years old child is buried in the western adventitious cist		• Deposition of burnt human remains brought inside the monument from elsewhere					
	• A 25 to 30-year-old woman deposited into the dolmen’s burial chamber through an opening created by perforating the covering slab The woman is accompanied by at least two jars		• Burial of a 4 to 10-year-old child in the chamber	• Violation of BB burials of the MV and relocation of human remains outside the chamber, north to the monument	• Perturbation of the BB burials of MVIII and violation of the BB burials of MVII with relocation of part of the human remains in the area between MVII and MVIII			• Violation of BB burials and relocation of human remains outside the chamber
	• Deposition of stones and faunal remains in the burial chamber covering the previously inhumated woman		• Removal of the skull and mandible of the infant	• Deposition of a fetus in the burial chamber of MV	• First accumulation of stones			• Deposition of a jar inside the chamber and original entrance sealed
EBA II	• Two new episodes of deposition of jars in the burial chamber of MXI; jars covered by stones and faunal remains Accumulation of stones around the monument Deposition of a jar in the southern adventitious cist • Perturbation of the EBA burial in the western adventitious cist	/	• Removal of the southern slab and deposition of some stones next to the northern slab			/	/	
EBA III	• Two episodes of deposition of jars in the burial chamber and accumulation of stones and faunal remains composing cairn III Jar deposition also next to south facade of the dolmen	/	/	• Deposition of jars in front of the southern facade of MV • Perturbation of BB burials in the cist MX • Breaking of the southern slab of MV		• Perturbation of BB burials and lighting of a fire inside the dolmen’s chamber Deposition of a first jar inside the monument • Cremation of human bones outside the dolmen • Construction of the adventitious cist on the southern side of the monument and inhumation of a child inside it • New stelae erected on the southern side of the monument, lighting of a fire in the adventitious cist, cremation of human bones north of the dolmen, and new perturbations of BB burials	/	/
EBA IV	• Deposition of jars in the burial chamber along with accumulation of stones and faunal remains Formation of cairn III continues and new depositions of jars next to the southern facade of the dolmen • Deposition of jars inside the chamber and of one human skull, accumulation of stones and faunal remains • Deposition of one jar in the dolmen’s chamber • Formation of cairn II filling the dolmen’s chamber and surrounding the monument • Formation of cairn I surrounding the south facade of the dolmen	/	/	• Formation of cairn II around dolmen MV It is exclusively composed of stones • Falling of the northern slab of dolmen MV • Formation of cairn I covering MV and MX	• Formation of a cairn covering the cists MVII and MVIII Fragments of jars from MVII	• Formation of cairn IV around the dolmen, composed of stones only • Lighting of a fire in the adventitious cist and in the chamber where human remains brought from elsewhere were burnt • Formation of cairn III around the dolmen, the southern facade in particular • Construction of a light hut west of the dolmen used in relation with the cultic activities Burning of the hut • Destruction of the cover slab of MVI • Formation of cairn II around the monument and deposition of jars on the eastern facade of the dolmen • Individual inhumation in the ground outside the dolmen Grave goods including bronze ornaments and weapons • Formation of cairn I and deposition of jars on the eastern facade of the dolmen	/	• Formation of a cairn around the cist Removal of the monument’s covering slab and deposition of stones inside the chamber

At the turn of 2nd millennium BC, during the phases EBA I and II, several previous BB burials were perturbed and the human remains and/or associated grave goods were dislocated outside the chamber, at the exterior of the monuments (MI, MIII, MV, MVII, MVIII, and MXI; see Table 1). New inhumations of a woman, children, and a fetus were also placed in monuments MXI, MIX, and MV. Jar depositions and accumulation of stones and faunal remains began during these two early phases, but became the predominant activities conducted at the site during the EBA III and IV phases, along with the construction of cairns (Table 1), which bear witness to the high level of engagement the protohistoric communities had with the megalithic monuments of the necropolis (Gallay and Chaix 1984; Gallay 1985, 1995, 2006, 2016; Besse et al. 2011). During the EBA IV period, in the western part of the *‘Petit-Chasseur I’* sector, a hut related to the cultic activities conducted in the monumental burials was built and then burnt. The repetitive violations and perturbations of the BB burials constitute an act of “desecration” of the funerary space (Gallay 1986, 1995; Besse et al. 2011). The new inhumations and the following ritual activities consisting in depositions of jars, faunal remains, and construction of cairns, however, show that the necropolis area soon regained a sacred status; this sacredness was related to the remembering of the important figures of society who were buried at the site (Gallay 1995). In the EBA II, III, and IV phases, the ancestors’ memory was passed on from one generation to the next until the EBA people started again to bury their dead in the area with a new type of non-megalithic individual tomb that consisted of a wooden container laid down in a pit lined with stone slabs (MVI, phase EBA IV) (Bocksberger 1978; Gallay 1995; Harrison and Heyd 2007). Grave goods included bronze personal ornaments and weapons, but no pottery (Bocksberger 1978; David-Elbiali 2000; Favre and Mottet 2011). At that time, the ancestors were more a concept to refer to rather than tangible families or individuals and were an anonymous but powerful force controlled by the EBA elite (Harrison and Heyd 2007).

**Fig. 3:**
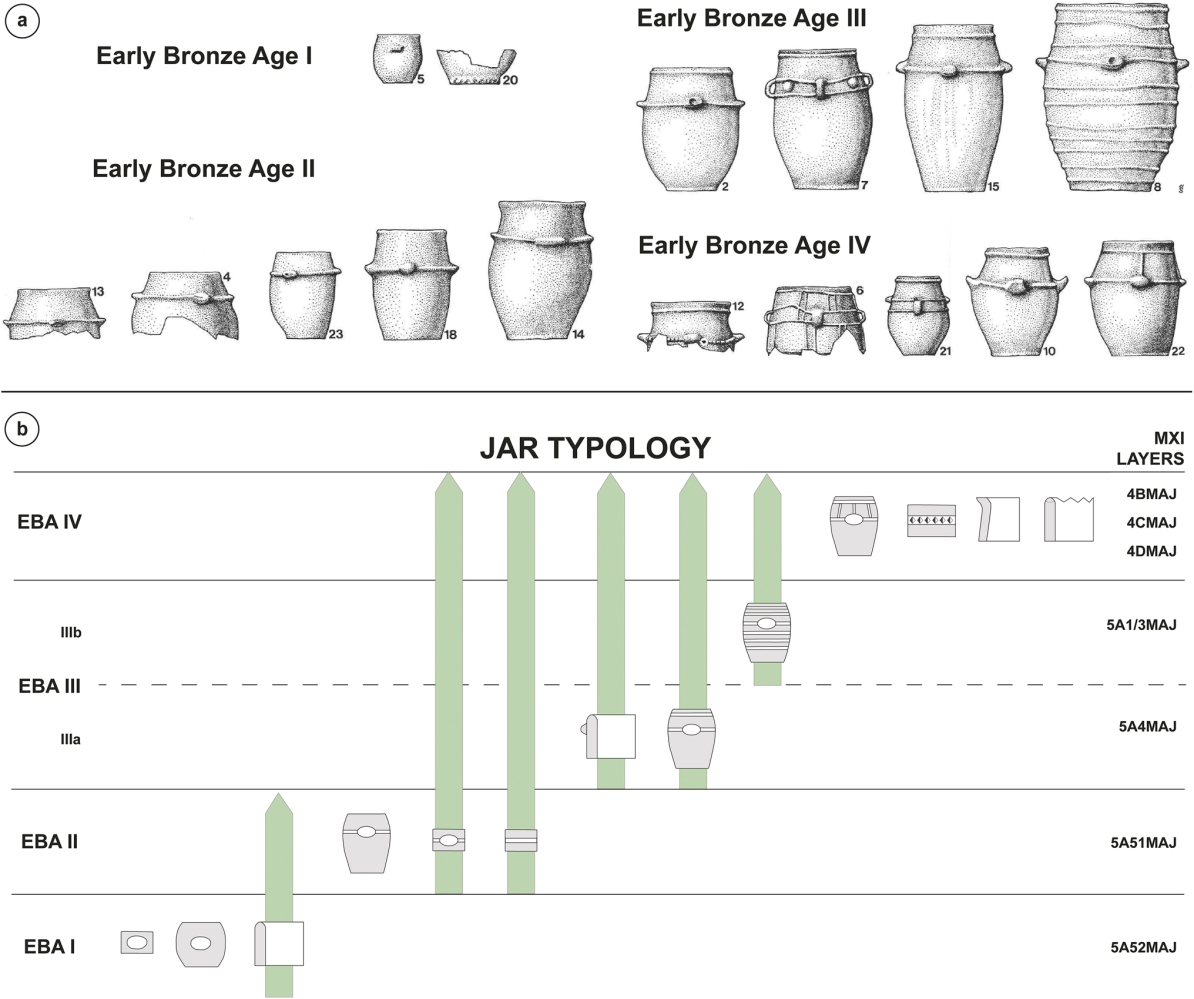
Stylistic features of the large storage containers related to the Early Bronze Age cultic activities at the Petit-Chasseur necropolis: **a** general characteristics of the jars (adapted from Gallay 1985) and **b** typo-chronology (adapted from Gallay and Chaix 1984)

Concerning the cultic activities in the previous megalithic tombs, the stylistic features of the jars deposited in the burial chambers and in the cairn structures changed throughout the four EBA phases, especially with regard to the number and position of cordons and the types of lugs (Fig. 3) (Gallay and Chaix 1984). The first jars were equipped with lugs simply placed on the vessel’s body, whereas starting from the EBA II the handles are positioned on a cordon. During the EBA III and IV another cordon is generally present under the rim, the lugs may be finger-impressed, and vertical strap handles appear in the ceramic assemblage. In the last EBA IV phase, finger-impressed and vertical cordons occur as well as finger-impressed rims. Few radiocarbon dates are available for the EBA horizon at the Petit-Chasseur necropolis: (1) 2468–1985 cal BC for the EBA I phase of MXI, (2) 2279–1916 cal BC for the EBA II-III of MXI, and (3) 2197–1745 cal BC for the EBA IV phase of MVI (Derenne et al. 2020). These dates overlap and, therefore, do not provide straightforward time spans for the different phases documented at the site. Hence, stratigraphy and ceramic typo-chronology are heretofore the only criteria for phase definition and periodization.

### The settlement sites

Seven settlement sites in the region yielded pottery material contemporaneous to the EBA cultic activities around the monumental tombs of the Petit-Chasseur necropolis (Carloni et al. 2020). The present study considers only six archaeological contexts: Sion *‘Petit-Chasseur III’, *Sion* ‘Sous-le-Scex’, *Vex* ‘Le Château’, *Salgesch* ‘Mörderstein’, *Rarogne* ‘Heidnischbühl II’,* and Naters* ‘Altersheim’* (Fig. 1; Table 2). The site of Ayent *‘Le Château’* was excluded as it did not provide enough material to carry out archaeometric analyses (Carloni et al. 2020). The ceramic typology of the procured pottery was addressed in detail in Carloni et al. (2020).

**Table 2:** List of Early Bronze Age settlements selected for the present study, with absolute chronology after Carloni et al. (2020), and corresponding phase of Petit-Chasseur necropolis. Radiocarbon dates are calibrated according to the curve IntCal13 (Reimer et al. 2013) and, whenever possible, modeled by Bayesian analysis (Bronk Ramsey 2009; 2017). Results reported with a two sigma (95.4%) certainty

Settlement site		Absolute chronology	Pottery stylistic features	Petit-Chasseur necropolis phase
1	Sion ‘Petit-Chasseur III’	Layer 4d: 2053–1306 cal BC	Cordoned jars, barrel-shaped jar decorated with button, cup	EBA III–IV
		Layer 4e: 2274–1772 cal BC	Cordoned jars, amphora, bowls, cups, regular and finger-impressed lugs, rounded and flattened rim fragments	EBA II?, III–IV
2	Sion ‘Sous-le-Scex’ “Sondage profond”	Layer 9: unknown	Flat base	EBA IV?
		Layer 10: unknown	Finger-impressed cordons	EBA IV
3	Vex ‘Le Château’	Layer 3: unknown	Finger-impressed cordons and rims, Roseaux-type cup	EBA IV
4	Salgesch ‘Mörderstein’	PHA16sup: 2275–2040 cal BC	Cordoned jars equipped with lugs and strap handles	EBA II–IV
5	Rarogne ‘Heidnischbühl II’	Layer 4: unknown	Cordoned jars, cordons under the rims, lug highly stretched upwards	EBA II–IV
6	Naters ‘Altersheim’	UT7: 2275–1955 cal BC	Cordoned jars and finger impressed lugs EBA II, III, and IV	EBA II–IV

**Fig. 4:**
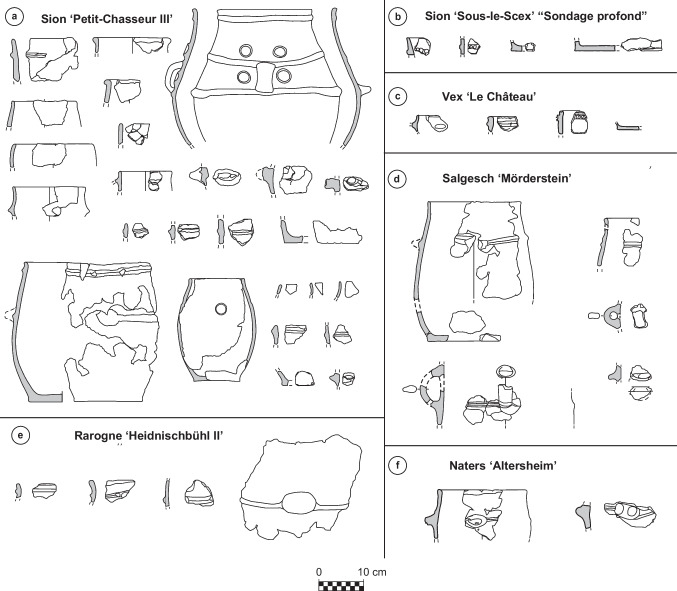
Typical pottery of the Early Bronze Age settlement sites of the Upper Rhône Valley: **a** Sion *‘Petit-Chasseur III’* (Favre and Mottet 1990); **b** Sion *‘Sous-le-Scex’* “Sondage profond” (Honegger 2011); **c** Vex *‘Le Chateau’* (David-Elbiali 1990); **d** Salgesch *‘Mörderstein’* (Gentizon-Haller et al. in press); **e** Rarogne *‘Heidnischbühl II’* (drawings: D. Carloni); **f** Naters *‘Altersheim’* (drawings: Ch. Gaudillière)

The settlement of Sion *‘Petit-Chasseur III’* was located on the sediments covering the FN dolmen MXII and the BB cist MXIII of the Petit-Chasseur necropolis (Fig. 2a) (Favre and Mottet 1990). The EBA horizon spans over two layers, 4e and 4d, that both yielded abundant charcoals, burnt clay nodules, potsherds and faunal remains; level 4d additionally hosted the remains of a hut (Favre and Mottet 2011). The ceramic assemblage documented at Sion *‘Petit-Chasseur III’* comprises a variety of shapes and handles (Fig. 4a): cordoned jars (layers 4e and 4d), barrel-shaped jar decorated with button (layer 4d), bowls (layer 4e) and cups (layers 4e and 4d), regular and finger-impressed lugs (layer 4e), and rounded and flattened rim fragments (layer 4e) (Favre and Mottet 2011; Carloni et al. 2020). These stylistic features of pottery suggest the occupation took place during the EBA III–IV period (Table 2) (Figs. 3b and 4a). Lugs placed on a cordon may suggest an EBA II occupation of the site; however, they may have belonged to a jar having a cordon under the rim and thus date to phase EBA III (Figs. 3b and 4a). Unfortunately, available radiocarbon dates do not provide a more detailed periodization (Bayesian modeling, level 4e dated to 2274–1772 cal BC and layer 4d to 2053–1306 cal BC; see Carloni et al. 2020).

The other five EBA settlement sites known in the region have more ephemeral occupations (Table 2) (Carloni et al. 2020). The closest one to the megalithic necropolis is the site of Sion *‘Sous-le-Scex’*, located ~ 1 km eastward (Brunier et al. 1986; Baudais et al. 1989). Only layers 10 and 9 found in the sector “Sondage profond” yielded proper EBA ceramics; the most ancient one, level 10, further hosted one posthole (Honegger 2011). A few diagnostic sherds bear finger-impressed cordons (Fig. 4b), which are typical of the EBA IV phase of Sion *‘Petit-Chasseur I’* (Fig. 3b) (Gallay and Chaix 1984; Carloni et al. 2020). Another domestic-like occupation in the vicinity of the megalithic necropolis is Vex *‘Le Château’,* located on the opposite side of the Rhône River and investigated by means of a 4-m^2^ test trench (Baudais et al. 1989). Layer 3 yielded a small EBA ceramic assemblage (David-Elbiali 1990; Carloni et al. 2020) that comprises one Roseaux-type cup and finger-impressed cordons and rims (Fig. 4c), similar to the EBA IV corpus of Sion *‘Petit-Chasseur I’* (Fig. 3b; Table 2). Three additional sites are located eastward in the URV. The PHA16sup level of the settlement of Salgesch *‘Mörderstein’* comprised hearths, pavings, and postholes and yielded cordoned jars equipped with lugs and strap handles (Fig. 4d) (Gentizon-Haller et al. in press). Stylistic features of ceramic containers display the typical traits of the EBA II, III, and IV material of Sion *‘Petit-Chasseur I’* (Fig. 3a, b). However, available radiocarbon dates point out the EBA occupation began at the turn of the 2nd millennium BC (Bayesian modeling: 2275–2040 cal BC, Table 2; Carloni et al. 2020). In regard to the site of Rarogne *‘Heidnischbühl II’,* it extended over 100 m^2^, but only a small area of layer 4 yielded EBA fragments of cordoned jars (Fig. 4e), which suggests the occupation of the area did not start before the EBA II phase (Fig. 3a, b; Table 2). The morphology of one lug placed on a jar is highly stretched upwards and is comparable to some big containers from the EBA IV phase. The lack of any ^14^C date does not allow for the definition of a precise time span (Carloni et al. 2020). The last and easternmost site of the corpus is Naters *‘Altersheim’*, which spans over 10 m^2^ and comprises pits and postholes (Mariéthoz 2005). Ceramic material includes cordoned jars and finger impressed lugs comparable to the EBA II, III, and IV phases (Fig. 4f; Table 2). However, one radiocarbon date from pit UT7 ranges 2275–1955 cal BC (Carloni et al. 2020).

## Geological background

**Fig. 5:**
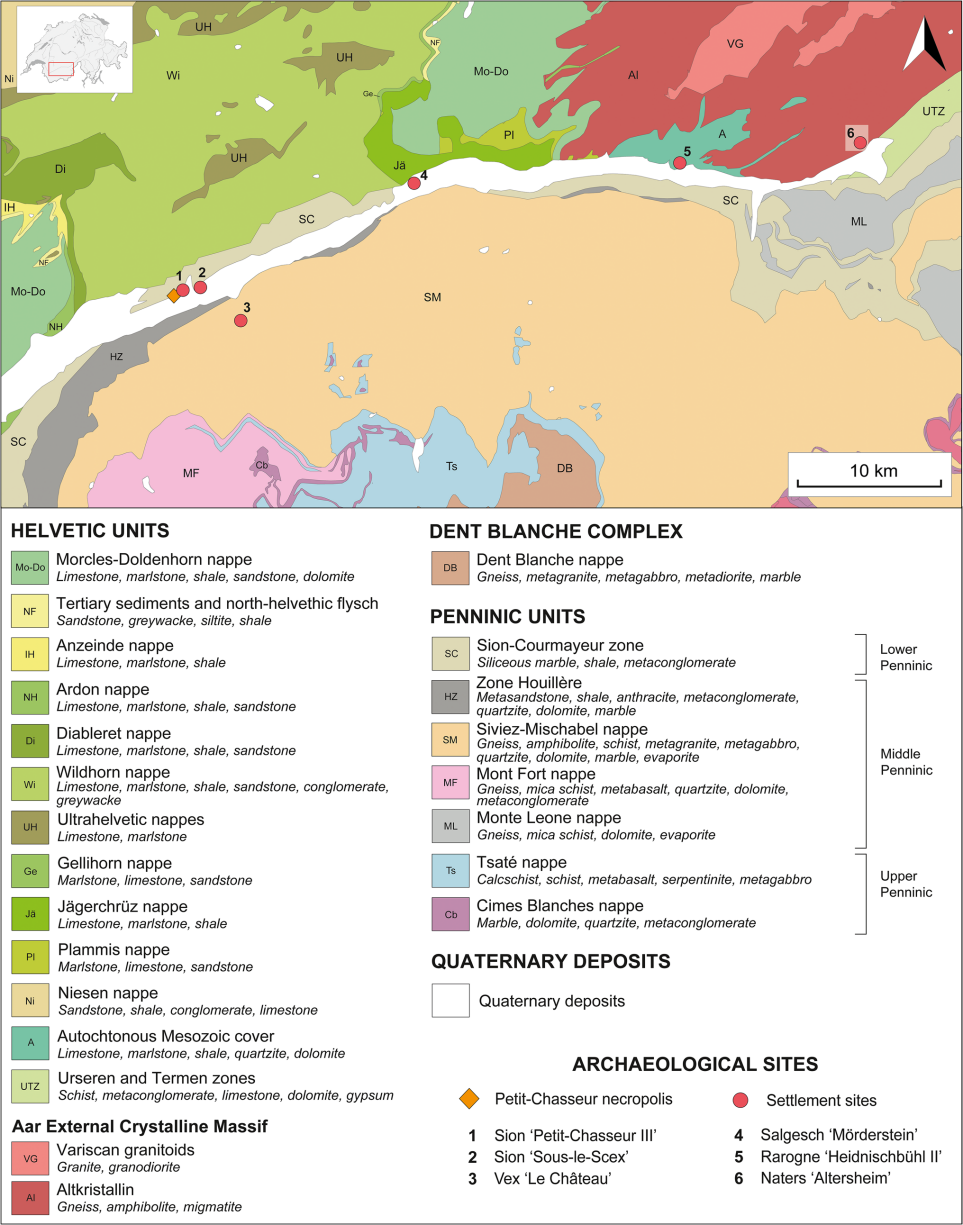
Litho-tectonic map of the research area with the main lithology outcropping in each nappe and the location of the archaeological sites. Adapted from data provided by the Swiss Federal Office of Topography (https://map.geo.admin.ch/)

The Western Alps formed as a result of the collision of the European continental margin and Adria microplate in the course of the Paleogene to Neogene orogenesis (Schmid et al. 1996, 2004; Handy et al. 2010). In the URV, three main groups of tectonic units are recognizable: the Dent Blanche Complex, the Penninic domain, and the Helvetic nappe stack (Fig. 5) (Stampfli 2001; Schmid et al. 2004; Pfiffner 2021). The Sesia-Dt Blanche nappe is the uppermost unit and comprises a crystalline basement of pre-Triassic age affected by greenschist metamorphism (Fig. 5) (Manzotti et al. 2014). The underlying Penninic nappe stack is composed of various lithospheric slivers belonging to distinct paleogeographic domains (Stampfli et al. 1998; Schmid et al. 2004). The upper Penninic unit is related to the Piedmont–Liguria ocean and accounts for ophiolitic and metasedimentary rocks resulted from metamorphism of eclogite, blueschist, and greenschist facies (Fig. 5) (Marthaler and Stampfli 1989; Schmid et al. 2004; Pleuger et al. 2005). The middle Penninic nappe represents the remains of the continental microplate that undergone blueschist (western part) to greenschist alpine metamorphism and consists of polymetamorphosed basement, former grabens occupied by Permo-Carboniferous fillings (metasandstone, conglomerate, shale, metavolcanic rocks), and a cover made of platform sediments (Fig. 5) (Thélin et al. 1993; Sartori and Marthaler 1994; Stampfli 2001; Bucher et al. 2003; Schmid et al. 2004; Sartori et al. 2007). Remnants of the narrow Valais basin compose the lower Penninic units made of Upper Cretaceous to Tertiary metaflysch affected by greenschist metamorphism (Fig. 5) (Stampfli et al. 1998; Schmid et al. 2004; Sartori et al. 2007). Finally, the Helvetic realm comprises a pre-Alpine European mylonitized and cataclasized crust uplifted and exhumed during the Alpine orogeny (Aar External Crystalline Massif; von Raumer et al. 1993, 2009; Burkhard and Sommaruga 1998; Stampfli 2001; Hettmann et al. 2009; Bellahsen et al. 2014) as well as sediments originated from the European continental margin (Pfiffner 1993; Herwegh and Pfiffner 2005; Sartori and Epard 2011). Both basement and cover have been affected by alpine low grade to greenschist metamorphism (Pfiffner 1993).

From a geomorphological point of view, the URV is characterized by glacial and gravitational landscapes originated from repetitive cycles of Quaternary glaciation and presence of steep slopes (Burri 1955; Ivy‐Ochs et al. 2008; Preusser et al. 2010; Sartori and Epard 2011; Valla et al. 2011; Ivy-Ochs 2015; Reynard et al. 2021). The short timespan since the final retreat of the large ice tongues (≥ 15,000 years; Ivy-Ochs 2015) as well as the cold and semi-arid climate conditions during the late Pleistocene and the Holocene hampered large-scale formation of clay deposits (Schlunegger and Hinderer 2003; Shakun and Carlson 2010; Goehring et al. 2011; Berthel et al. 2012; Schwörer et al. 2014). Possible clay procurement areas count for limited alluvial accumulations and torrential deposits of the Rhône River and its tributaries, lacustrine deposits, and pedogenized till, colluvial, and loess deposits (Reynolds 1971; Velde 1992; Velde and Meunier 2008; Galán and Ferrell 2013; Stalder 2015; Šegvić et al. 2018; Roux 2019).

## Materials and methods

### Materials

This study builds on the characterization of domestic pottery from EBA settlement sites which are contemporary to the cultic activities at the Petit-Chasseur sites (Bocksberger 1976, 1978; Gallay and Chaix 1984; Gallay 1989, 1995). The *Office cantonal d’Archéologie* and *Musée d’Histoire du Valais* in Sion host archaeological findings from EBA settlement sites. Our sampling strategy took into account the chronology, location, shape, and macroscopic characteristics of ceramic paste. The acquired sample set accounts for a total 46 ceramic fragments from settlement sites contemporary to EBA cultic activities around the megalithic monuments of the Petit-Chasseur necropolis. The ceramic assemblage of Sion *‘Petit-Chasseur III’* (PC81-PC100, Supplementary material 1) is represented by 20 samples dating to EBA phases III–IV, possibly to EBA II as well. They account for three EBA III-IV cordoned jars, one ovoidal amphora, 14 body fragments, and two daub fragments. The latter were selected to obtain a more comprehensive view on the choice and manipulation of clays by EBA communities of the URV. As far as samples from other domestic-like occupations are concerned, the four body fragments of Sion *‘Sous-le-Scex’* “Sondage profond” (SS01-SS04) and one potsherd from Vex *“‘Le Château’”* date to the EBA IV phase (VX01), whereas the deposit of Salgesch *‘Mörderstein’* is represented by one EBA II cordoned jar, one EBA II-IV cordon, and body fragments with no further phase periodization (SM04-SM06 and SM08, Supplementary material 1). The sample set of Rarogne *‘Heidnischbühl II’* comprises one EBA II–IV cordoned potsherd, one EBA III–IV cordoned jar, one EBA IV rim fragment, and four sherds attributed to the same EBA IV cordoned jar (RH01-RH07, Supplementary material 1). Finally, for the body fragments of Naters *‘Altersheim’* from pit UT7, no further stratigraphic information or typological features allow for phase attribution (NA01-NA10, Supplementary material 1). Further information regarding the sampled material may be found in Supplementary material 1.

Previously published data on EBA jars from the Petit-Chasseur necropolis (total 62 samples; Carloni et al. 2021) have been considered solely for comparison purposes (Supplementary material 1). These results were used in this contribution to facilitate the correlation with potsherds recovered from domestic-like contexts. Sampled material were selected from the assemblages related to the main events reported for the history of MXI (Table 1): depositions of jars in the burial chambers (EBA phases II, III, and IV) and in correspondence to the south facade of the dolmen (EBA phases III and IV). In addition, samples were taken from ceramics linked to the deposition of jars around MV (EBA phase III), MVII (EBA phases III or IV), and MVI (EBA phase IV). As outlined in Table 1, the cultic activities around MIII, MVIII, MIX, and MX did not involve the abandonment of ceramic containers; no evidence of rituals was found in the stratigraphic sequence of MII (Bocksberger 1978).

### Methods

The ceramics were characterized by means of multiple microscopic and spectroscopic techniques. Petrographic analysis was executed using optical microscopy (OM), whereas mineralogical investigation was carried out through X-ray diffractometry (XRD) and scanning electron microscopy coupled with energy-dispersive spectrometry (SEM–EDS). Finally, geochemistry was acquired by inductively coupled plasma mass spectrometry (ICP-MS) and laser ablation inductively coupled plasma mass spectrometry (LA-ICP-MS).

#### Polarization microscopy

A Leica Leitz DM-RXP polarizing microscope was used to analyze 45 ceramic thin sections from settlement sites (Supplementary material 1). The study was done at the University of Geneva and consisted in the description of the principal petrographic features of ceramic paste (matrix, voids, inclusions) following the guidelines of Whitbread (1989) and Quinn (2013) and clustered into fabrics (Supplementary material 2). The size of aplastic inclusions was recorded according to the Udden-Wentworth (U-W) grain size scale revised by Terry and Goff (2014).

#### X-ray diffraction

The mineralogical examination was carried out on 36 powdered samples (Supplementary material 1) using a Bruker D8 Advanced diffractometer at the Department of Geosciences of Texas Tech University. Diffraction patterns were acquired from 3 to 70°2*Θ* with a step size of 0.02° and time of 1.8 s/step with a generator setup of U = 40 kV and I = 40 mA and interpreted with the help of the Bruker EVA software suite and the PDF4 database implemented by the International Centre for Diffraction Data.

#### Scanning electron microscope

An in-depth investigation of the matrix mineralogy and microtexture was executed on a subset of 13 ceramic carbon-coated thin sections by means of SEM–EDS (Supplementary material 1). The analysis of the ceramics from the Petit-Chasseur necropolis and the two daub samples from Sion *‘Petit-Chasseur III”* was carried out using a Zeiss Crossbeam 540 apparatus equipped with the EDS installed at the Microscopy Center of the College of Arts and Sciences of Texas Tech University. The characterization of the ceramics from settlement sites was done using the FEI QEMSCAN^®^ Quanta 650F facility of the University of Geneva. Analyses were performed under high vacuum (acceleration voltage of 15–20 kV) with two silicon drift energy-dispersive X-ray detectors from Bruker. The secondary and backscattered electron modes were used to capture images of the ceramic matrices. Acquired EDS spectra were quantified in a standardless mode using the Bruker ESPRIT software and were normalized to 100%.

#### Geochemistry

The Bureau Veritas Laboratories in Vancouver (Canada) performed ICP-MS geochemical analyses on 32 samples (Supplementary material 1). Samples weighted ~ 5–9 g and were prepared by grinding, mixing with a lithium metaborate/tetraborate flux, and dissolving in nitric acid. Digestion in Aqua Regia was additionally carried out to detect the presence of rare and refractory elements. Weight loss after ignition at 1000 °C was carefully registered and determined the loss on ignition value. Due to their light weight, 10 samples were analyzed by LA-ICP-MS at the Bureau Veritas Laboratories in Perth, Australia (Supplementary material 1). Analyses were performed on fused discs prepared from ~ 1.7–4 g of starting material. Repeated analyses of different sample aliquots indicated a relative standard deviation of ± 0.3% and ± 0.5% for major and trace elements, respectively.

The raw elemental data obtained by means of ICP-MS/LA-ICP-MS (Supplementary material 3) were log_10_-transformed to standardize the available dataset to inter-comparable values (e.g. Baxter 1995; Aruga 2003; Hall 2004; Baxter and Freestone 2006; Papachristodoulou et al. 2010; Tanasi et al. 2017). The STATISTICA 13 software package was then used to perform Principal component analysis (PCA) and unveil compositional similarities within the sample set. PCA induces a smaller number of artificial variables called principal components (PCs) to reduce a multidimensional data and generate a plot constructed using the first two (or three) PC variables (e.g. Beier and Mommsen 1994; Papachristodoulou et al. 2010). Consequently, ceramics with similar composition are shown in the form of agglomerated points in the plot (e.g. Mommsen 2001; Šegvić et al. 2012, 2016; Carloni et al. 2021).

## Results

### Ceramic petrography

**Fig. 6:**
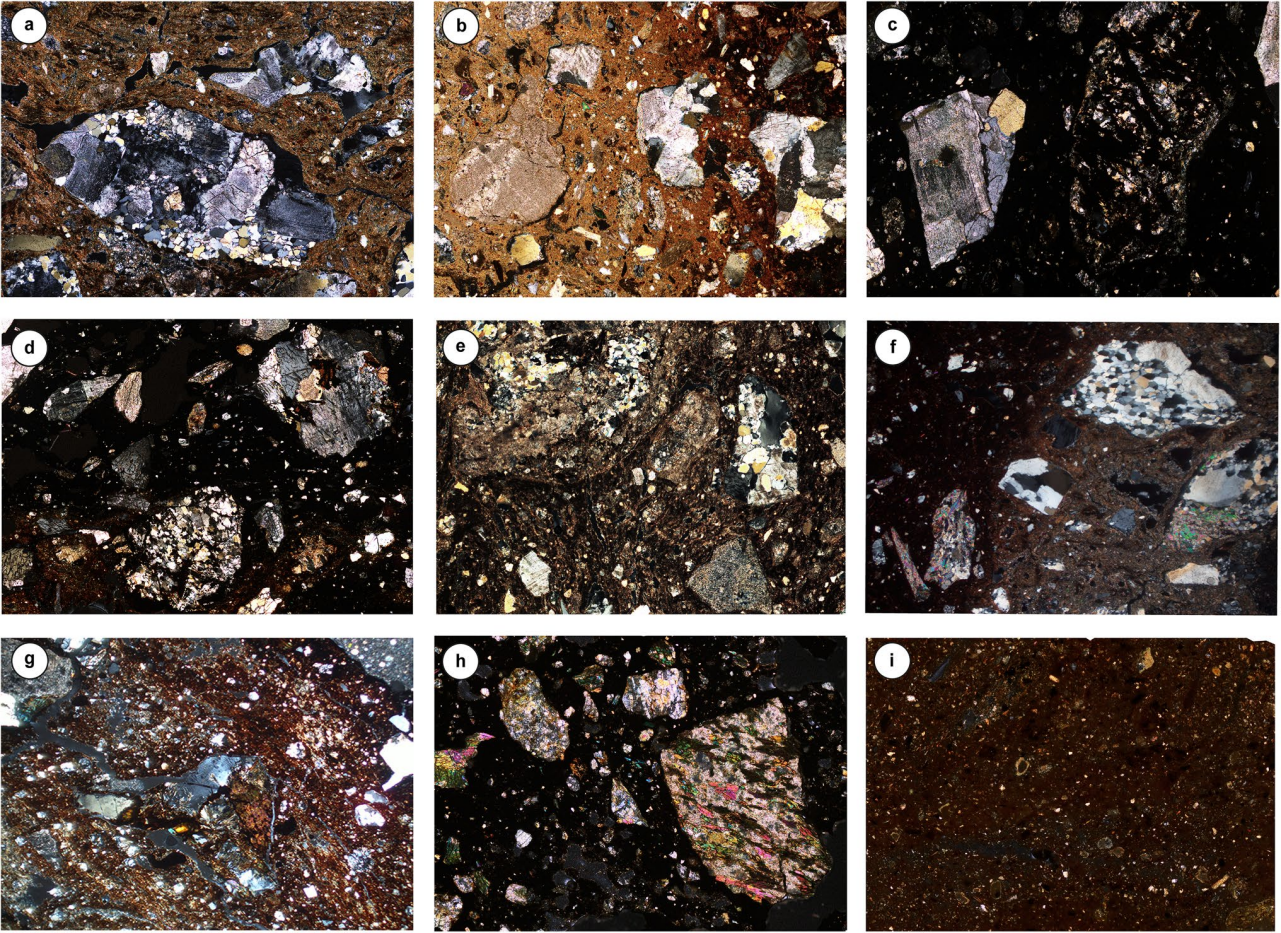
Microphotographs of selected Petit-Chasseur pottery representative of the distinct fabrics: **a** granite var. 1 (PC11); **b** granite var. 3 (PC100); **c** fine-grained granite rich in Fe oxide var. 1 (NA03); **d** fine-grained granite rich in Fe-oxide var. 2 (PC99); **e** weathered granite (PC56); **f** quartz–feldspar gneiss var. 2 (PC48); **g** amphibole gneiss var. 1 (PC17); **h** amphibolite (RH07); **i** allochem (PC85). Image width 5.3 mm

An optical microscopy examination of ceramic paste (matrix, void, inclusions) allowed a definition of six ceramic fabrics (Table 3, Fig. 6). Their detailed description can be found in Supplementary material 2. The type and morphometry of aplastic inclusions strongly distinguish analyzed pottery and therefore play a key role in fabric classification. Internal variability within the fabric, whenever relevant, was referred to as “variant” (e.g., fabric 1—granite, var. 1, var. 2, and var. 3—see Table 3). Previously published data on EBA jars from the Petit-Chasseur necropolis (Supplementary material 1; Carloni et al. 2021) is provided in Table 3 and Supplementary material 2 solely for comparison.

**Table 3:** Description of the fabric; variant 1, 2, and 3 (var. 1, var. 2, var. 3) are distinguished by the presence or absence of additional discriminant lithologies and/or differences among the dominant-to-common fraction. Approximate amount of clasts was determined by visual estimation using common comparison charts. The following abbreviations are used to indicate the grain size distribution (GSD): unimodal (U), bimodal (BI), trimodal (TRI), and polymodal (POLY)

					Aplastic inclusions					
	Fabric		Matrix	Voids	% clast	Dominant 50–90%	Frequent-common 15–50%	Few-rare 0.5–15%	GSD	Max Ø
**1**	**Granite**	**Var. 1** Samples: PC11, PC12, PC20, PC23, PC25, PC34, PC35, PC38, PC47, PC69, PC70	73–82%	3–7%	13–20%	Biotite-rich granite, granite	Fine-grained granite with secondary calcite	Quartz, feldspar, biotite, white mica, sedimentary rocks (carbonate mudstone, Fe-rich carbonate rock, sandstone with volcanic rock fragments), low-grade metamorphic rocks (quartz schist, mica schist, chlorite schist)	BI, TRI	4 mm
		**Var. 2** Samples: PC49, PC52, SM04, SM08, SS01, SS02, SS03	65–87%	3–10%	10–25%	Biotite-rich granite or granite		Biotite, quartz, low-grade metamorphic rocks (cataclasite, mica schist)	BI, TRI	8 mm
		**Var. 3** Samples: PC96, PC97, PC100	75–80%	5%	15–20%	Granite	Micritic limestone	Carbonate mudstone, quartz, low-grade metamorphic rocks (mica schist, chlorite schist), white mica	POLY	4.3 mm
**2**	**Fine-grained granite rich in** **Fe-oxide**	**Var. 1** Samples: NA02, NA03, NA04, NA05, NA06, NA07, NA08, NA10, SS04	77–87%	3–5%	10–20%	Fine-grained granite with Fe-oxide	Granite	Quartz, biotite, white mica, metamorphic rocks (mica schist, quartz-feldspar gneiss)	BI, TRI	5.8 mm
		**Var. 2** Samples: PC21, PC82, PC88, PC91, PC92, PC98, PC99, NA01, NA09, SM05, SM06, VX01	70–87%	3–5%	10–25%	Biotite-rich granite or granite	Fine-grained granite with Fe-oxide	Quartz, feldspar, biotite, white mica, carbonate, low-grade metamorphic rocks (mica schist, chlorite schist), phosphate	BI, TRI, POLY	6 mm
**3**	**Weathered granite** Samples: PC56, PC57		70–75%	5–10%	20%	Weathered granite (secondary calcite)		Quartz, feldspar, biotite, white mica, granite	TRI	6.5 mm
**4**	**Quartz-feldspar gneiss**	**Var. 1** Samples: PC89, PC90, PC94	70–77%	3–5%	20–25%	Quartz-feldspar gneiss	Granite	Quartz, biotite, white mica	BI, TRI	4.3 mm
		**Var. 2** Samples: PC40, PC48, PC66, PC81, PC83	68–82%	3–7%	10–25%	Quartz-feldspar gneiss	Fine-grained granite with secondary calcite	Quartz, white mica, low-grade metamorphic rocks (mica schist)	BI, TRI	5 mm
		**Var. 3** Samples: PC39, PC54, PC59, PC62, PC63, PC84, PC87 PC95	73–85%	3–7%	10–20%	Biotite-rich granite or granite	Quartz-feldspar gneiss	Quartz, feldspar, biotite, white mica, fine-grained granite with Fe-oxide, low-grade metamorphic rocks (mica schist), carbonate	BI, TRI	6.8 mm
**5**	**Amphibole-rich rocks**	**Var. 1** Samples: PC16, PC17, PC32	78–82%	3–7%	15%	Amphibole gneiss		Quartz, feldspar, amphibole, biotite, white mica, sedimentary rocks (limestone), fine-grained granite, fine-grained granite with secondary calcite	BI, TRI	5.4 mm
		**Var. 2** Samples: PC30, PC31, PC33	75–87%	3–7%	10–15%	Biotite-rich granite		Amphibole gneiss, quartz, feldspar, biotite, white mica, sedimentary rocks (limestone), low-grade metamorphic rocks (mica schist)	BI, TRI	3.2 mm
		**Var. 3** Samples: RH01, RH02, RH03, RH04, RH05, RH06, RH07	65–75%	5%	20–30%	Amphibolite	Fine-grained granite with Fe-oxide	Quartz, granite, white mica, low-grade metamorphic rocks (mica schist, quartz schist)	BI, TRI, POLY	4.9 mm
**6**	**Allochem** Samples: PC85, PC86		92–94%	1–3%	5%	Allochem	Quartz	White mica, mica schist	U	0.7 mm

#### Fabric 1: granite

This fabric was observed in samples from the Petit-Chasseur necropolis and the domestic-like occupations of Sion *‘Petit-Chasseur III’*, Salgesch *‘Mörderstein’*, and *‘Sous-le-Scex’* “Sondage profond” (total 21 samples; Table 3). Ceramic pastes of this kind have heterogeneous non-calcareous matrix showing slight to moderate optical activity (Fig. 6a). The color of the groundmass is yellowish‐to reddish‐brown with only rare instances of black. The voids are mega and macro channels or planar voids and macro and meso vughs, oriented either randomly or in a parallel configuration.

Regarding aplastic inclusions, all the samples of this fabric bear coarse silt- to gravel‐sized particles derived from the granite–granodiorite–quartz diorite family (Table 3, Supplementary material 2). Ceramics of variant 1 also contain sand-sized rounded inclusions of fine-grained granite with post-depositional calcite infills, whereas samples of variant 3 are rich in carbonate sedimentary rocks (Table 3, Fig. 6b, Supplementary material 2). Inclusions are poorly sorted, equant, and elongated in shape, with angular to subangular edges (Fig. 6a, b). The grain size distribution in this group is bimodal, trimodal, or polymodal (Supplementary Material 2).

#### Fabric 2: fine-grained granite with Fe-oxide

The samples of this group belong to the ceramic assemblages of the Petit-Chasseur necropolis, Sion *‘Petit-Chasseur III*,” Sion *‘Sous-le-Scex”* “Sondage profond”, Naters *‘Altersheim’*, Salgesch *‘Mörderstein’*, and Vex *‘Le Château’* (total 21 samples; Table 3). Ceramic matrix is heterogeneous or homogeneous, yellowish-, reddish-, and blackish-brown colored, with a slight to moderate degree of optical activity. Voids are mega- and macro-sized in the case of channels, and macro- and meso-sized in the case of vughs. Orientation is random or parallel to the margins of samples (Supplementary material 2).

The association of fine-grained granite with Fe-oxide and granite inclusions characterizes this fabric (Table 3, Fig. 6c, d, Supplementary material 2). Inclusions are sand to gravel in size, are equant or elongate in shape, and have angular to subrounded edges. The grain size distribution observed in this fabric is bimodal, trimodal, or polymodal (Supplementary material 2).

#### Fabric 3: weathered granite

This fabric was exclusively documented in two samples of the Petit-Chasseur necropolis (Table 3). They display a heterogeneous and mostly yellowish-brown matrix that has a slight to moderate degree of optical activity. Voids are macro channels and meso vughs, randomly oriented or parallel to the pot’s walls (Supplementary material 2). Aplastic inclusions consist of subrounded to subangular equant and elongate inclusions of highly weathered igneous rocks (Fig. 6e). A trimodal grain size distribution features the samples of this group (Supplementary material 2).

#### Fabric 4: quartz–feldspar gneiss

Samples of this fabric are part of the findings of the Petit-Chasseur necropolis and Sion *‘Petit-Chasseur III’* (total 16 samples; Table 3). Their ceramic groundmass is heterogeneous, brown, reddish‐ and yellowish-brown as well as black in color, showing slight to moderate optical activity. The paste presents macro and meso channels and macro to micro vughs occurring in random or parallel configurations (Supplementary material 2).

Presence of quartz–feldspar gneiss particles defines this group (Fig. 6f). Gneiss inclusions range from gravel to sand size and are characterized by equant and elongate shapes with angular to rounded edges. In the ceramic paste of this group’s samples, particles of the granite–granodiorite–quartz diorite family are additionally present (Table 3). Granite inclusions are gravel to sand in size and equant to elongate in shape with angular to subrounded edges. Fine-grained granite grains are accompanied by secondary calcite and are sand-sized and rounded. The grain size distribution is bimodal to trimodal (Supplementary material 2).

#### Fabric 5: amphibole-rich rocks

This group comprises thirteen samples belonging to the ceramic assemblage of the Petit-Chasseur necropolis and Rarogne *‘Heidnischbühl II’* (Table 3). The matrix is heterogeneous and displays slight to moderate optical activity; its color is reddish-brown, dark yellowish-brown-, and dark grayish-brown to black-colored. Macro- to meso-sized channel voids occur in the paste along to meso vughs, both with a random or parallel orientation (Supplementary material 2).

Aplastic inclusions assemblage is dominated by the presence of high-grade metamorphic rocks of amphibolite facies, whose shape is equant and elongate with rounded to subangular edges. Amphibole gneiss inclusions are gravel- to medium sand-sized and their edges are angular to subangular; their presence features variant 1 and 2 of the fabric (Fig. 6g). Particles are rich in albite and quartz, hornblende, and accessory minerals such as epidote and titanite. In the samples displaying the fabric characteristics of variant 2, granite is present as well. These inclusions are gravel-to medium sand-sized, equant and elongate, with angular to subrounded edges. Variant 3 bears gravel- to medium sand-sized particles of amphibolite, whose mineralogy accounts for hornblende, albite or alkali feldspar, ilmenite, and titanite (Fig. 6h). In addition, samples of variant 3 host sand-sized particles of fine-grained granite with Fe-oxide, whose shape is equant or elongate and the edges are rounded to subangular. A bimodal to polymodal grain size distribution features the aplastic inclusion assemblage of the pastes of this group (Supplementary material 2).

#### Fabric 6: allochem

Only two daub samples from clay from Sion *‘Petit-Chasseur III’* display these fabric characteristics (Table 3). The groundmass is homogeneous, calcareous to certain degree, and yellowish-brown in color. The degree of optical activity is slight. Macro‐ and meso‐vughs are present and are randomly oriented. The paste contains inclusions of carbonate allochems (Fig. 6i) composed of micritic calcite and lack a well-defined internal structure. Their size ranges between coarse silt and coarse sand with a unimodal grain size distribution (Supplementary material 2).

### XRD mineralogy

X-ray diffraction analysis provided further insights into the mineralogical composition of URV EBA pottery. Previously published data on EBA jars from the Petit-Chasseur necropolis (Supplementary material 1; Carloni et al. 2021) have been provided in Table 4 for comparatively purposes only. Acquired dataset shows quartz and feldspars are ubiquitous phases whereas other non-phyllosilicates were only sporadically found (Table 4). Calcite is present in the samples of fabric 1 var. 3 (samples PC96, PC97, PC100) and carbonate allochem-rich samples (fabric 6, samples PC85, PC86), and is sporadically reported in the other petrography-based groups (fabrics 1–4, samples PC11, PC56, PC83, PC87, SM08, SS04). An Fe-oxide, most likely hematite, was measured in samples characterized by particles of granite (fabric 1 var. 1, 2, 3), gneiss (fabric 4), and carbonate allochems (fabric 6). Epidote and amphibole were identified in amphibolite-bearing pottery (fabric 5); the latter was further on found in ceramics marked by fabrics 1, 2, 4, and 6 (Table 4). Zeolite minerals were exclusively documented in samples belonging to fabric 1 (SS01, SS02, PC96, PC97, PC100), while minor phases such as garnet and sulfides were identified only rarely (Table 4).

**Table 4:** XRD mineralogy of the ceramics analyzed, by fabric

Fabric	Sample	XRD
1: Granite var. 1	PC11	Chl, 10 Å mica, Qtz, Kfs, Ab, Mc, Cal, Hem
	PC38	Chl, 10 Å mica, Qtz, Kfs, Ab, Hem
	PC69	Chl, 10 Å mica, Qtz, Kfs, Mc, Ab, Hem
1: Granite var. 2	PC49	10 Å mica, Qtz, Mc, Ab
	SM04	Vrm, 10 Å mica, Qtz, Kfs, Ab
	SM08	Vrm, 10 Å mica, Qtz, Kfs, Ab, Cal
	SS01	10 Å mica, I-S, Zeo, Pg, Qtz, Kfs, Ab, Am
	SS02	10 Å mica, I-S, Zeo, Pg, Ber, Qtz, Kfs, Ab, Am
1: Granite var. 3	PC96	Chl, 10 Å mica, Zeo, Sm, Qtz, Kfs, Ab, Cal, Grt
	PC97	10 Å mica, Zeo, Sm, Qtz, Kfs, Ab, Cal
	PC100	Chl, 10 Å mica, Zeo, Sm, Tos, Qtz, Kfs, Ab, Cal, Dol, Grt
2: Fine-grained granite var. 1	NA03	10 Å mica, Qtz, Kfs, Ab, Hem
	NA04	10 Å mica, Qtz, Kfs, Ab
	NA05	10 Å mica, Sm, Qtz, Kfs, Ab
	NA06	10 Å mica, Qtz, Kfs, Ab, Hem
	NA08	10 Å mica, Qtz, Kfs, Ab
2: Fine-grained granite var. 2	NA01	10 Å mica, Qtz, Kfs, Ab, Py
	PC82	Chl, 10 Å mica, Qtz, Kfs, Ab, Hem
	PC84	Chl, Vrm, 10 Å mica, Qtz, Kfs, Ab, Hem
	PC92	10 Å mica, Qtz, Kfs, Ab
	PC99	Vrm, 10 Å mica, Qtz, Kfs, Ab
	SM05	10 Å mica, Qtz, Kfs, Ab, Hem
	SM06	10 Å mica, Qtz, Kfs, Ab
	SS04	10 Å mica, I-S, Pg, Qtz, Kfs, Ab, Cal, Am
	VX01	10 Å mica, Qtz, Kfs, Ab
3: Weathered granite	PC56	Chl, 10 Å mica, Qtz, Kfs, Ab, Cal, Hem
4: Quartz-feldspar gneiss var. 1	PC89	Vrm, 10 Å mica, Qtz, Kfs, Ab, Am
	PC90	Chl, 10 Å mica, Qtz, Kfs, Ab, Am
4: Quartz-feldspar gneiss var. 2	PC48	Chl, 10 Å mica, Qtz, Kfs, Ab
	PC66	Chl, 10 Å mica, Qtz, Kfs, Mc, Ab, Hem
	PC81	10 Å mica, Qtz, Kfs, Ab, Hem
	PC83	10 Å mica, Qtz, Kfs, Ab, Cal, Hem
4: Quartz-feldspar gneiss var. 3	PC87	Chl, 10 Å mica, Qtz, Kfs, Ab, Cal, Hem
	PC59	Chl, 10 Å mica, Qtz, Kfs, Mc, Ab, Hem
	PC63	10 Å mica, Qtz, Mc, Ab
5: Amphibole-rich rocks	RH01	10 Å mica, Qtz, Ab, Am
	RH02	10 Å mica, Qtz, Ab, Am
	RH03	10 Å mica, Qtz, Ab, Am
	RH04	Chl, Vrm, 10 Å mica, Qtz, Ab, Am, Ep
	RH05	Chl, Vrm, 10 Å mica, Qtz, Ab, Am, Ep
	RH06	Chl, Vrm, 10 Å mica, Qtz, Kfs, Ab, Am, Ep
	RH07	Chl, Vrm, 10 Å mica, Qtz, Kfs, Ab, Am, Ep
6: Allochem	PC85	Chl, 10 Å mica, Qtz, Kfs, Ab, Cal, Am, Hem
	PC86	Chl, 10 Å mica, Qtz, Kfs, Ab, An, Cal, Di, Hem

*Ab* albite; *Am* amphibole; *An* anorthite; *Ber* bermanite; *Cal* calcite; *Chl* chlorite; *Di* diopside; *Dol* dolomite; *Ep* epidote; *Grt* garnet; *Hem* hematite; *I-S* illite–smectite; *Kfs* K-feldspar; *Mc* microcline; *Pg* paragonite; *Py* pyrite; *Qtz* quartz; *Sm* smectite; *Tos* tosudite; *Vrm* vermiculite; *Zeo* zeolite

10-Å phyllosilicates (fine-grained mica and illite) were identified in the entire sample set based on their characteristic *d*_001_ peaks ranging between 9.5 Å and 10.0 Å (10 Å mica, Table 4) (Moore and Reynolds 1997). In addition, the 14 Å phyllosilicates were found common in analyzed ceramics. Presence of chlorite was revealed by the *d*_001_, *d*_003_, and *d*_004_ basal reflections at ~ 14.2 Å, ~ 18.7 Å, and ~ 25.11 Å respectively (Moore and Reynolds 1997; Meunier 2005). Chlorite occurs in most of analyzed potsherds (Table 4). On the contrary, vermiculites—diagnostic reflections at ~ 14.5 Å and ~ 7.5–8.2 Å (Campos et al. 2009)—were found in few potsherds with fabrics 1, 2, 4, and 5 (samples PC84, PC89, PC99, RH04-07, SM04, SM08). Finally, smectite emerges in several samples belonging to fabrics 1 and 2 (Table 4) as a discrete phase (*d*_001_ reflections at ~ 15 Å in the air-dried conditions and shifts to 16–17 Å after ethylene glycol treatment—samples PC96, PC97, PC100, NA05, Moore and Reynolds 1997). Mixed-layer illite–smectite is also reported (*d*_001_ at 12–14 Å which migrates to lower angle after ethylene glycol treatment—samples SS01, SS02, SS04, Moore and Reynolds 1997; Meunier 2005; Borchardt 2018).

### SEM–EDS mineralogy of ceramic matrix

**Fig. 7:**
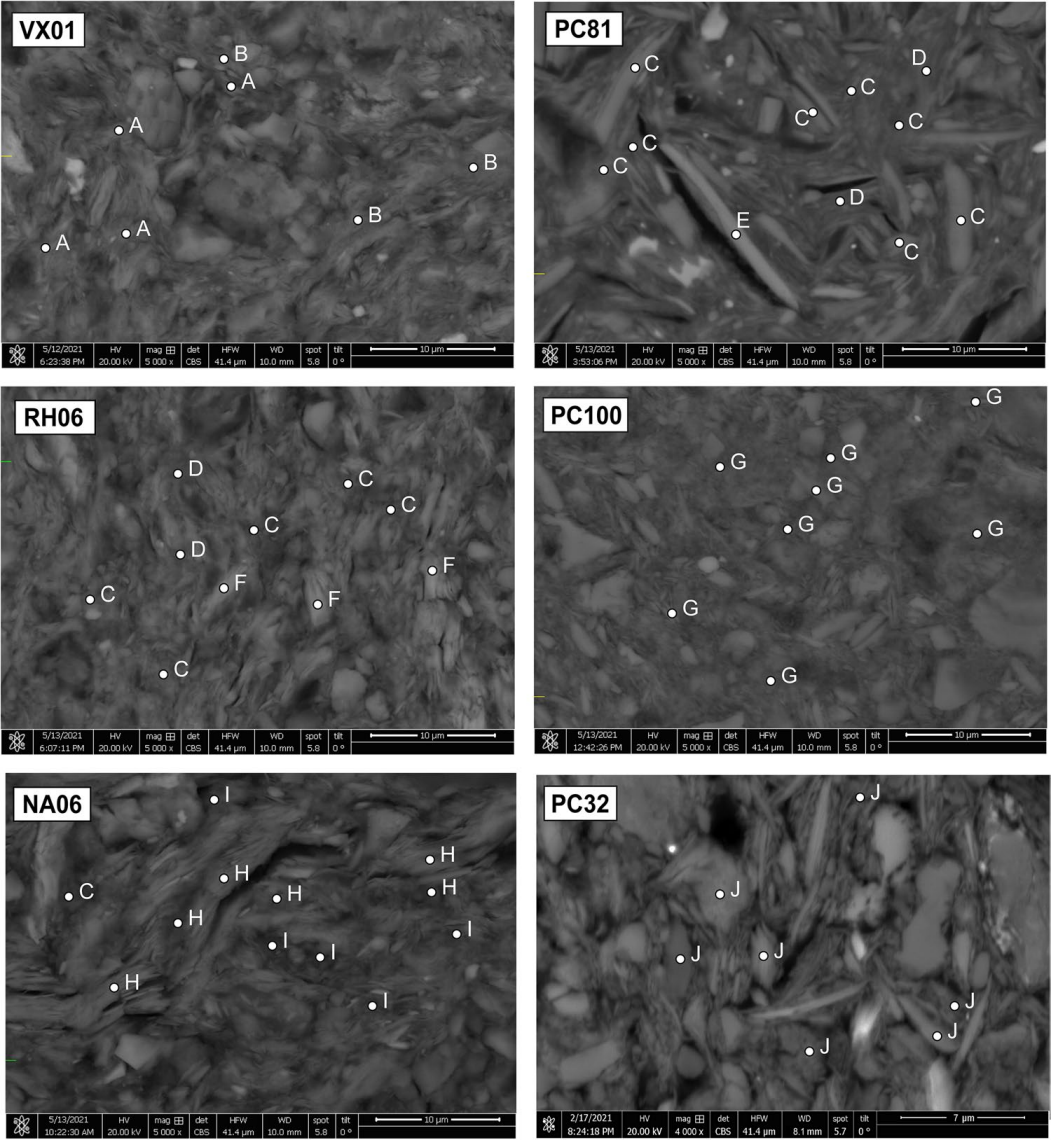
Backscattered images of selected ceramic matrix. Exemplary EDS spectra (A, B, C, etc.) may be found in Table 5

SEM–EDS analysis allowed insights in the composition of the ceramic groundmass (Fig. 7; Table 5). The majority of analyzed pottery from settlement sites have a groundmass consisting of illite and Fe-illite (~ 5–16% FeO), partially affected by smectitization (illite–smectite interlayers: Si/Al ratio ~ 2.5). Similar matrix composition was reported by Carloni et al. (2021) for most of EBA jars from the Petit-Chasseur necropolis (PC66, PC48, PC56, results available in Table 5). Potsherds PC83, PC84, and RH06 feature presence of mica-vermiculite (> 9% FeO, ~ 7–9% MgO, Table 5) and detrital mica and chlorite. The vermiculitization is likely related to biotite alteration (Moon et al. 1994; Murakami et al. 2003). The clay substrate of potsherds PC100 and PC85 is somewhat different; the former is exclusively made of Mg-illite (~ 6–12% MgO) while the latter has a very heterogeneous composition made of illite, smectite, chlorite, and muscovite (Fig. 7; Table 5). Regarding muscovite-like minerals (Fig. 7; Table 5), K-poor mica platelets (Si/Al ratio ~ 1/1 and K_2_O ~ 4–6%) feature the groundmass of NA05, NA06, SM05, and SS04 (Table 5). This 2:1 clay mineral may be affected by illitization (Si/Al ratio ~ 1.3, samples SM05 and SS04; Table 5) and may be Fe-enriched (~ 5–18% FeO in samples NA06 and SS04, Table 5). Illite and Fe-illite were also reported as accessory phases in muscovitic groundmass. This composition was also documented in EBA jars from the Petit-Chasseur necropolis (e.g., PC63, Carloni et al. 2021; results available in Table 5). Lastly, the potsherds’ matrix from settlement sites does not show similarities with potsherd PC32 from the Petit-Chasseur necropolis whose groundmass is made of vermiculite (Carloni et al. 2021; results available in Table 5).

**Table 5:** EDS chemistry of the ceramic matrix of analyzed samples. Backscattered images of VX01, PC81, RH06, PC100, NA06, PC32 can be found in Fig. 7

Sample	Fabric	Mineral	Spectrum	SiO_2_	Al_2_O_3_	K_2_O	FeO	MgO	CaO	Na_2_O	TiO_2_	Total
PC66	4	Illite	245	53.9	27.2	7.0	6.3	3.6	1.6	-	0.3	100%
		Chlorite	248	38.1	23.5	2.0	20.0	12.5	0.9	-	2.8	100%
PC84	4	Illite	359	55.8	29.1	3.6	4.7	5.6	0.8	0.4	-	100%
		Muscovite	352	49.3	31.0	9.3	5.0	3.8	0.3	0.6	0.6	100%
		Mica-vermiculite	363	37.1	25.1	2.4	21.2	12.6	1.1	0.5	-	100%
PC48	4	Illite–smectite	314	69.6	17.8	4.5	3.2	3.8	0.8	-	0.3	100%
		Weathered mica	309	53.4	26.3	11.9	5.1	3.3	-	-	-	100%
		Smectite	285	69.8	17.6	5.6	3.0	3.2	0.8	-	-	100%
VX01	2	Illite (A)	300	53.9	35.2	3.9	3.6	1.4	1.0	0.6	0.4	100%
		Illite (A)	303	58.7	30.3	3.2	4.3	1.4	1.4	0.2	0.4	100%
		Smectite (B)	301	60.1	27.6	5.3	2.5	1.9	1.5	0.3	0.8	100%
PC56	3	Fe-rich illite	568	54.0	27.4	6.5	5.9	4.6	1.1	-	0.6	100.1%
		Illite–smectite	571	46.5	20.5	3.1	14.3	13.4	1.3	-	0.9	100%
PC81	4	Fe-rich illite (C)	368	49.0	30.5	7.4	6.6	4.6	0.6	0.7	0.6	100%
		Illite–smectite (D)	374	58.0	25.9	3.3	4.3	5.4	2.2	0.6	0.3	100%
		Chlorite (E)	366	33.6	25.5	1.6	22.4	14.6	0.8	0.2	1.2	100%
SS02	1	Fe-rich illite	279	50.8	33.1	2.3	6.9	2.0	2.7	0.8	1.3	100%
		Fe-rich illite	283	48.9	33.0	2.5	9.3	2.1	2.0	0.4	1.8	100%
		Illite	285	51.5	34.5	3.7	4.3	3.9	0.6	0.4	0.4	100%
SM04	1	Fe-rich illite	414	51.8	30.3	5.8	6.8	2.7	0.9	1.0	0.9	100%
		Fe-rich illite	415	45.7	24.5	5.4	14.6	5.5	1.3	0.5	2.2	100%
		Illite–smectite	425	63.5	16.0	2.9	9.4	2.5	2.4	0.4	2.1	100%
PC83	4	Fe-rich illite	389	47.7	30.3	6.2	7.4	4.7	2.5	0.6	0.4	100%
		Illite	388	53.7	27.1	7.2	4.7	2.2	1.6	2.77	0.2	100%
		Mica-vermiculite	385	39.8	23.7	1.7	17.4	15.6	0.7	0.6	0.2	100%
		Chlorite	382	30.3	27.1	0.9	18.3	22.5	0.5	0.2	0.1	100%
RH06	5	Fe-rich illite (C)	409	52.0	31.9	4.3	6.5	2.9	0.8	0.5	0.8	100%
		Fe-rich illite (C)	408	45.1	30.6	4.4	12.3	4.8	1.6	0.5	0.4	100%
		Mica-vermiculite (F)	403	39.6	27.0	3.1	20.0	7.1	2.0	0.5	0.5	100%
		Illite–smectite (D)	412	56.5	26.8	4.4	7.5	2.7	0.9	0.4	0.6	100%
PC100	1	Mg-rich illite (G)	349	51.0	30.8	5.8	4.3	6.2	0.7	0.4	0.7	100%
		Mg-rich illite (G)	346	48.6	26.5	4.8	5.2	12.8	1.0	0.6	0.4	100%
PC85	6	Illite	118	49.5	40.6	0.8	6.2	1.2	-	-	1.5	100%
		Smectite	115	64.5	19.6	11.9	1.2	0.4	-	1.6	-	100%
		Chlorite	112	29.7	22.3	-	19.1	28.8	-	-	-	100%
		Muscovite	116	47.5	38.5	10.0	2.4	1.3	-	0.4	-	100%
		Calcite and clay mixed analysis	114	35.6	22.6	-	19.8	-	21.9	-	-	100%
PC63	4	K-poor mica	454	57.4	30.5	4.6	4.9	1.2	2.1	0.4	1.6	100%
		Illite	462	65.4	23.2	3.8	4.7	0.9	1.7	0.3	-	100%
		Muscovite	451	50.6	37.2	9.2	1.8	0.8	-	0.5	-	100.1%
		Smectite	461	74.9	16.9	2.1	3.6	1.0	1.4	0.2	-	100.1%
NA05	2	K-poor mica	331	47.6	39.9	5.2	4.0	1.6	0.3	0.4	0.9	100%
		Illite	335	56.1	32.9	5.2	3.2	1.3	0.3	0.4	0.4	100%
		Muscovite	328	47.3	39.9	6.0	4.4	1.3	0.1	1.3	0.6	100%
SM05	2	K-poor mica	435	47.1	38.1	4.4	4.4	2.6	1.4	1.1	0.8	100%
		K-poor mica, illitization	434	48.5	36.4	4.0	5.5	2.5	1.3	0.8	0.9	100%
		Illite	437	50.1	34.2	4.8	4.9	1.7	1.8	1.1	1.2	100%
		Fe-rich illite	438	47.9	33.7	3.9	7.6	2.9	1.7	1.0	1.2	100%
		Chlorite	428	29.5	29.8	1.6	29.3	7.4	0.5	0.4	1.2	100%
NA06	2	Muscovite (H)	308	46.9	37.7	7.5	4.4	2.2	0.1	0.3	0.6	100%
		K-poor and Fe-rich mica (I)	313	40.6	42.3	5.2	7.8	2.5	0.6	0.4	0.5	100%
		K-poor and Fe-rich mica (I)	324	39.2	37.1	5.0	13.7	3.3	0.5	0.3	0.5	100%
		Fe-rich illite (C)	318	57.2	24.6	2.6	12.0	2.7	0.5	0.3	0.1	100%
SS04	2	K-poor and Fe-rich mica	268	41.1	35.8	2.2	14.4	2.3	2.7	0.4	1.2	100%
		K-poor and Fe-rich mica, illitization	269	39.9	31.4	4.2	18.5	0.9	3.2	0.4	1.4	100%
		Fe-rich illite	273	44.8	29.5	1.2	16.0	0.2	3.1	2.8	2.4	100%
PC32	5	Vermiculitized mica (J)	512	56.8	19.2	8.2	3.7	10.6	1.4	-	-	100%
		Vermiculitized mica (J)	511	58.6	21.7	7.4	4.5	6.1	1.7	-	-	100%

**Fig. 8:**
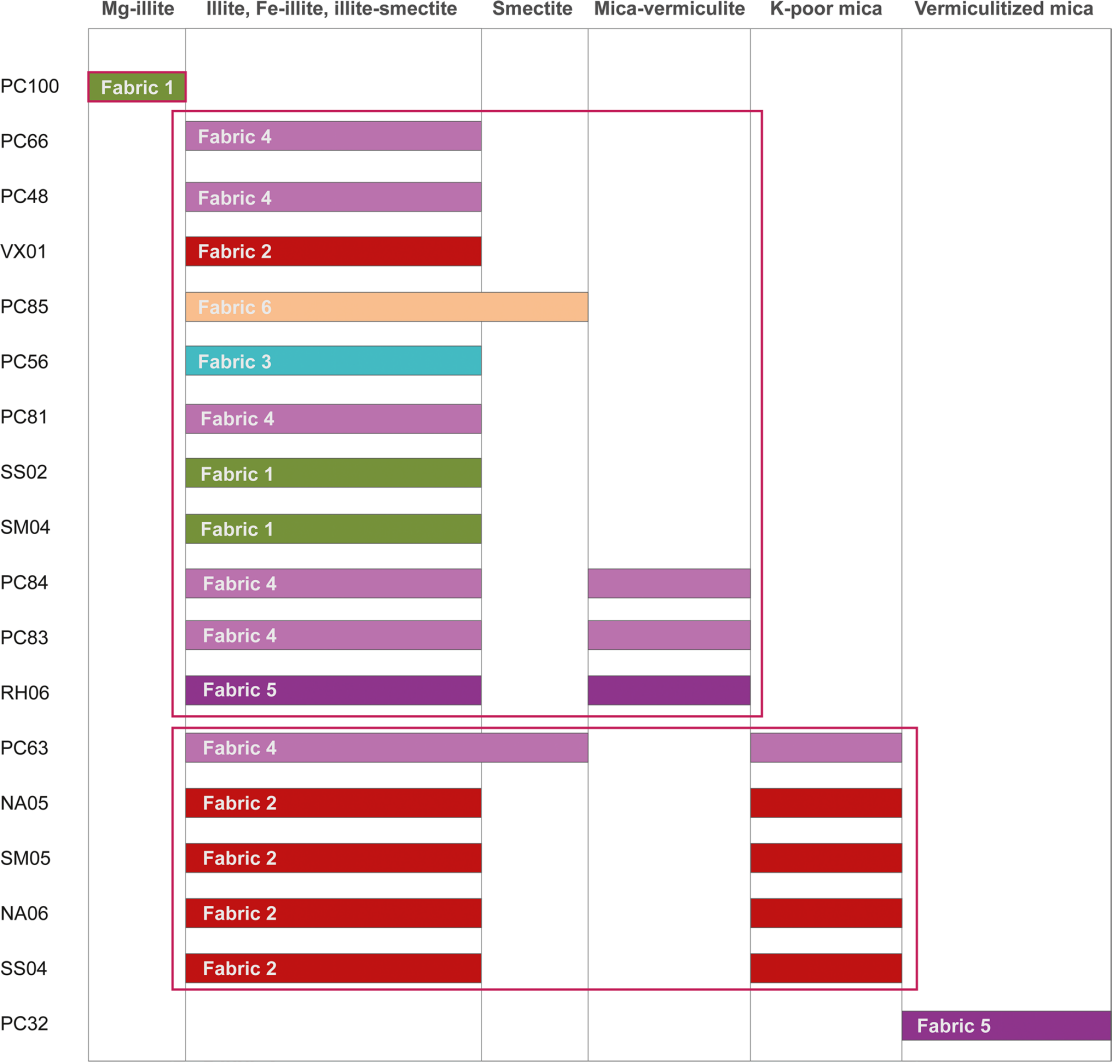
Association of clay mineralogy and fabric in analyzed ceramics

The URV pottery matrix, based on SEM–EDS characterization, is either illite or mica dominated (Fig. 7). The former accounts for Mg-illite (posherd PC100) and illite to Fe-illite groundmass (potsherds PC48, PC56, PC81, PC83, PC84, PC85, RH06, SM04, SS02, and VX01, Fig. 7), whose shape and size are not visible. The latter encompasses pottery samples whose matrix is characterized by abundance K-poor mica, occurring in the form of platelets of various size (NA06, Fig. 7) and, to a lesser extent, illite or Fe-illite (NA05, NA06, PC63, SM05, SS04, Fig. 7). We were not able to correlate our findings on the clay substrate mineralogy (Table 5; Fig. 8) with the type of aplastic inclusions as samples belonging to the same petrographic group readily show various matrix compositions.

### Elemental composition

Raw geochemical data (Supplementary material 3) were studied to identify the element concentration patterns characteristic of EBA ceramics. The majority of analyzed pottery consists of SiO_2_ ~ 50–70 wt%, Al_2_O_3_ ~ 11–20 wt%, CaO ~ 0.5–5 wt%, Fe_2_O_3_ ~ 2–6 wt%, and MgO ~ 0.5–4 wt%. Comparatively high CaO concentrations ~ 7–9 wt% mark the composition of three potsherds (PC96, PC97, PC100) and two daub fragments (PC85, PC86) from Sion *‘Petit-Chasseur III’*. In addition, the samples from Rarogne *‘Heidnischbühl II’* (RH01–RH07) have CaO ~ 0.5–5 wt% coupled with Fe_2_O_3_ ~ 6–8 wt%. Concerning trace element content, it ranges around ~ 0.1–0.6 wt% and the total amount of rare earth elements (REE) varies considerably, from 127 to 391 ppm (Supplementary material 3). Finally, all the samples have a U/Th ratio of 0.12–0.6, except for the ceramics from Naters *‘Altersheim’* whose ratio ranges between 1.4 and 2.8.

**Fig. 9:**
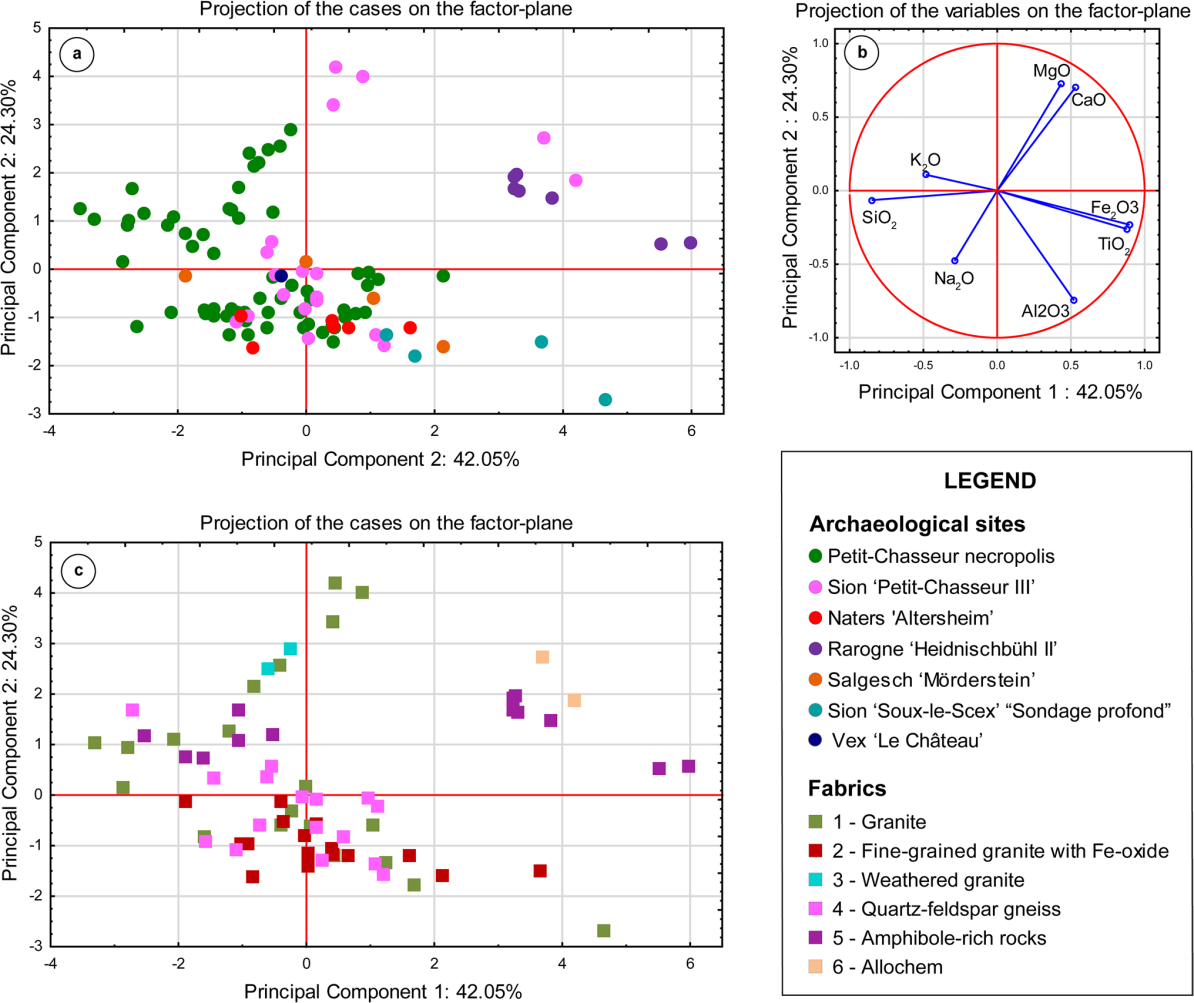
Statistical treatment of geochemical data of analyzed pottery: **a** and **b** PCA performed considering major and minor element content (Supplementary material 3), principal component biplot; **c** same principal component biplot of **a** with mention of the fabric

The PCA based on major and minor element contents of pottery from both the Petit-Chasseur necropolis and settlement sites displayed the first three components accounting for 80.46% of the total variance (42.05%, 24.30%, and 14.11%, respectively) (Fig. 9). Point agglomerations resulted from various amounts of SiO_2_/K_2_O, CaO/MgO, and Fe_2_O_3_/Ti_2_O, which controls the projection areas. Ceramics from the megalithic cemetery and settlement sites showed analogous composition (Fig. 9a). This is related to similarities in the amounts of felsic/mafic components determined by the types of aplastic inclusions occurring in the ceramic paste and fabric classification (Fig. 9b; Table 3). As a matter of fact, pottery characterized by the occurrence of various quartz and feldspar-based rocks (fabrics 1, 2, and 4) cluster in the same projection area. However, compresence of weathered granite particles (secondary calcite infillings, fabrics 1 var. 1, 3, and 4 var. 2), limestone inclusions (fabric 1 var. 3), or amphibole gneiss (fabric 5 var. 1 and 2) differed from the main projection field and projected as outliers (Fig. 9b). Finally, ceramic pastes bearing amphibolite and allochem inclusions revealed to be enriched in CaO and/or Fe_2_O_3_/Ti_2_O (Fig. 9b).

**Fig. 10:**
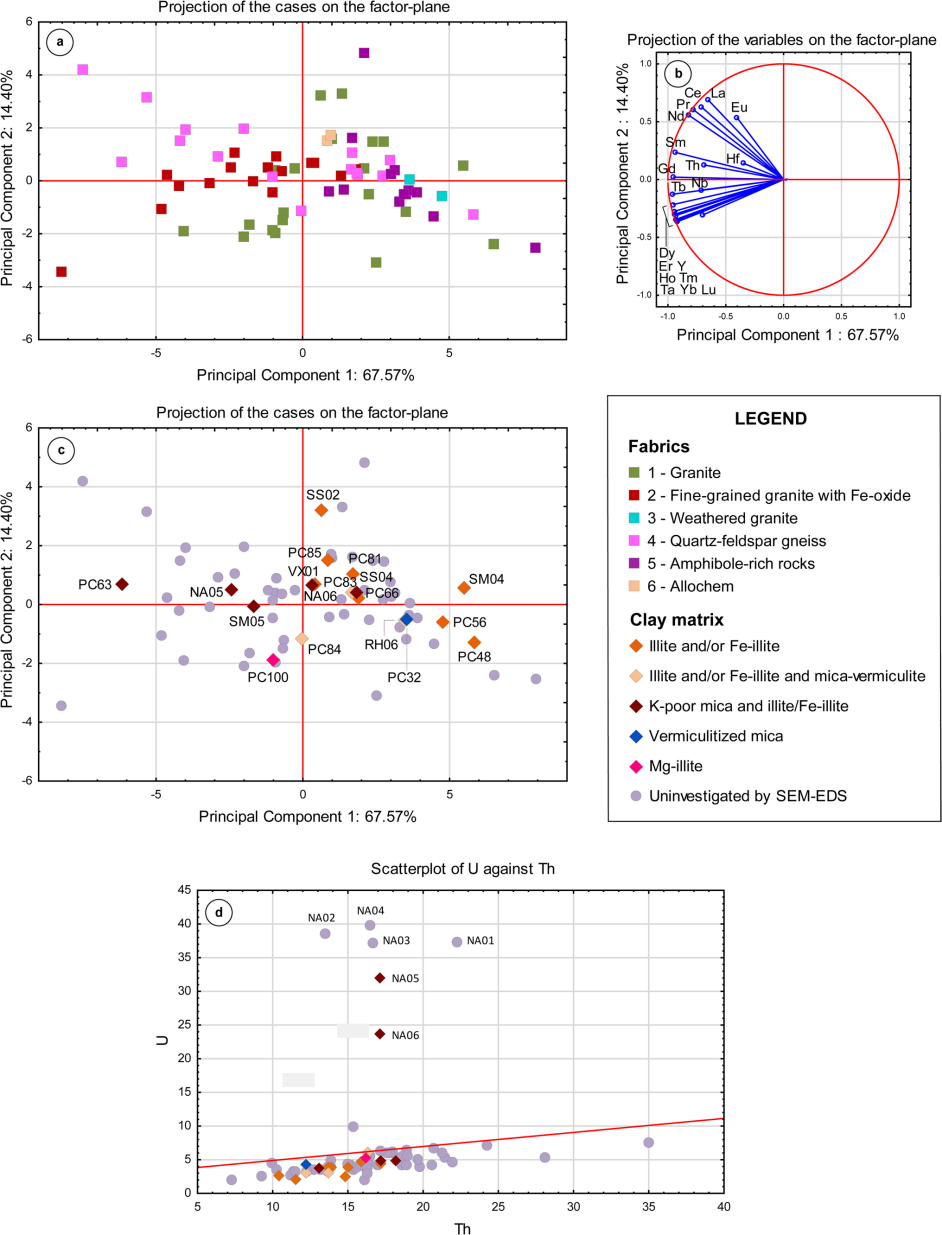
Statistical treatment of geochemical data of analyzed pottery: **a** and **b** PCA performed considering REE and trace element budget of the samples for which the lithological assemblage is known, principal component biplot; **c** same principal component biplot of **a** with location on the factor-plane of the samples for which the SEM–EDS analysis has been executed; **d** bivariate plot of U versus Th (values expressed in ppm)

The PCA based on REE concentrations of pottery from both the Petit-Chasseur necropolis and settlement sites showed the first three PC components accounting for 90.18% of the total variance (67.57%, 14.40%, and 8.21%, respectively; Fig. 10). Total amount of REE and relative ratios of light rare-earth elements (LREE) and heavy light rare-earth elements (HREE) (Fig. 10b), which however did not correlate with kind of aplastic inclusions occurring in the ceramic paste (Fig. 10a). This can be inferred based on the projection areas of samples displaying the same fabric characteristics and is particularly evident in the various REE loads of samples belonging to fabrics 1 and 4 (Fig. 10a). Concerning the possible correlation with matrix mineralogy (Fig. 10b, c), one can observe that ceramics whose groundmass is mainly composed of muscovite-like phases tend to be enriched in both LREE and HREE (PC63 and NA05), whereas illitic matrices generally display lower REE concentrations (Fig. 10c). Samples whose matrix is marked by the co-presence of mica platelets and Fe-illite show an intermediate REE concentrations (samples SM05, NA06, and SS04, Fig. 10c); notably, samples SS04 and SM05 are comparatively REE-poor due to illitization of clay substrate (Table 5). In addition, samples in which illite–smectite was detected by SEM–EDS (Table 5) tend to be relatively impoverished in REE content (samples PC48, PC56, SM04, Fig. 10b, c) as well as sample PC32 whose matrix consists of vermiculitized mica (Fig. 10b, c).

Finally, the U/Th bivariate plot shows that matrix mineralogy does not entirely control U and Th content (Fig. 10d). The ceramics from Naters *‘Altersheim’* drift apart as outliers due to the high U, even considering that their clay mineralogy is similar to that of PC63, SM05, and SS04 (Fig. 10d; Table 5). The rest of the sample set has similar U/Th ratios (< 1; Supplementary material 3) but differs in terms of Th content. This results in two areas of point agglomeration: one with Th concentration between 7 and 14 ppm and a second one with Th between ~ 15–22 ppm (Fig. 10d).

## Discussion

### Raw material choices

Archaeometric analyses revealed clear resemblance between vases from the necropolis and settlement sites in terms of petrography, mineralogy, and geochemistry (“Results” section). In other words, jars deposited as offerings around the megalithic monuments and domestic pottery display similar fabric characteristics, mineralogical assemblage, and scatter distributions in elemental-based biplots (Tables 3, 4, 5; Figs. 6, 7, 8, 9, 10). In this section, we discuss acquired compositional data with respect to the raw material choices and paste preparation recipes.

Particles’ morphometric characteristics, bimodal to polymodal grain-size distribution, and lack of correlation between aplastic inclusions and clay substrate (“Ceramic petrography” and “Elemental composition” sections) suggest tempering practices for ceramics of fabrics 1 through 5 (Table 3) (Velde and Druc 1998; Quinn 2013; Heimann and Maggetti 2014; Fowler et al. 2019; Eramo 2020). Tempering of raw clay is beneficial for paste workability, for reduction of shrinkage, for facilitation of the drying process, and for ensuring strength and toughness of the ceramic body (Rye 1976; Arnold 1985; Rice 1987; Velde and Druc 1998; Tite et al. 2001; Quinn 2013; Roux 2019; Eramo 2020). Furthermore, it has been demonstrated that presence of intrusive rocks in the ceramic paste (fabrics 1–3; Table 3) increases thermal conductivity of the ceramic body and does not hamper the vessels’ resistance to thermal shock (Hein et al. 2008). Abundance of quartz in quartz-feldspar gneiss inclusions of fabric 4 (“Fabric 4: quartz–feldspar gneiss” section) likely hampered the use of these vessels as cooking pots due to high coefficient of thermal expansion of quartz (Tite et al. 2001). The pottery rich in amphibole-rich fragments (fabric 5; Table 3) is thought to withstand well the thermal stress due to high thermal stability of amphibole (Arndt and Häberle 1973; Jenkins 1983; Rapp 2009; Tribaudino et al. 2010). At present, there is however no research on influence of quartz-feldspar gneiss/amphibole gneiss/amphibolite tempers on properties of archaeological ceramics. Concerning vessels’ shape, usage of intrusive and metamorphic rocks as temper material is documented in pots of various forms and sizes (Fig. 4) irrespectively of their function—e.g., food and beverage preparation or consumption, storage, transport (Rice 1987; Skibo 2013). Hence, EBA potters of the URV might have preferred a range of temper material with no aimed influence on vessels’ thermal or mechanical performance.

With regard to temper material procurement, granite, quartz-feldspar gneiss, and amphibole-rich rocks outcrop in the Aar External Crystalline Massif and the Penninic unit of Switzerland (Fig. 5; “Geological background” section) (Sartori et al. 2006; Hettmann et al. 2009; von Tscharner et al. 2016) which are both found in the morainic deposits related to the Rhône Glacier (Burri 1958; Sartori and Epard 2011; Stalder 2015). The till sediment largely composes the Quaternary cover of the URV and is available in the vicinity of archaeological sites considered in the present study. However, the Rhône Glacier’s deposits account for 50–90% of carbonates (Burri 1958; Sartori and Epard 2011; Stalder 2015), which in turn never exceed 10% of aplastic inclusions in the ceramic paste of fabrics 1 through 5 (Table 3). This indicates that the original composition of coarse sediment used as temper material was presumably manipulated by manually removing carbonate inclusions, which is a technique widely used by traditional potters for processing ceramic raw material (e.g., Michelaki et al. 2015; Roux 2019). Selection of granite particles from the Rhône Glacier’s deposits as temper material has been documented for the 4th and 3rd millennia BC ceramic production in Switzerland, where potters intentionally avoided carbonates and calcareous clay for pottery production no matter of their abundance in the local environment (Nungässer and Maggetti 1992; Maggetti 2009; Besse et al. 2019; Stapfer et al. 2019). In addition to lithological sorting, potters might have also subjected the granite/gneiss particles to fragmentation to reduce the size of coarser particles (Roux 2019). Regarding the samples of fabric 1 var. 3 (Table 3, “Fabric 1: granite” section), they include both granite and limestone and witness the use of the Rhône Glacier’s deposits as temper material without further manipulation. Another case where the original source of temper material was not manipulated can be found in the ceramics from Naters *‘Altersheim’* (fabric 2, Table 3). This may be due to the fact that the morainic and colluvial deposits located in the vicinity of the archaeological site were devoid of sedimentary rocks (Moulin 2014).

Based on the macroscopic appearance of granite/quartz-feldspar gneiss/amphibole gneiss, another inference can be made on potters’ capability to discriminate these types of rocks. Millimeter-sized (biotite-rich) granite, quartz-feldspar gneiss, and amphibole gneiss particles are hardly distinguishable. It is therefore probable that EBA potters of the URV simply selected rock particles composed of dark- and light-colored silicates from a source they largely had at their disposal—i.e., Rhône Glacier deposits—and whose behavior in the ceramic manufacturing process was known by tradition. This would explain the omnipresence of granite inclusions in the analyzed ceramics and the heterogeneity in pastes’ lithological assemblage (see fabric variants; Table 3; “Ceramic petrography” section). An exception is made for amphibolite, which can be easily recognized even in millimeter-sized particles by the abundance of dark-colored hornblende. In this case, potters therefore might have preferred those particles displaying larger amounts of dark-colored silicates in the local Rhône Glacier’s deposits or colluvial deposits.

Concerning the clay used in pottery manufacturing, compositional data suggests a predominant use of sources enriched in 10 Å phyllosilicates. SEM–EDS analysis revealed that the matrices of analyzed ceramics are essentially twofold: illite-based or muscovite-based. Illite and mica platelets represent optimal clay material for pottery manufacturing because of their poor hydration and moderate plasticity, which favor a quick drying process and low rates of weight and volume loss (Rice 1987; Velde and Druc 1998; Velde and Meunier 2008). The two types of clay minerals usually originate from distinct kinds of weathering: mostly mechanical in the case of muscovitic clay and predominantly chemical for illitic clay (Rich and Obenshain 1955; Meunier and Velde 1976; Chamley 1989; Velde 1992; Coppin et al. 2002; Bergaya and Lagaly 2006; Velde and Meunier 2008; Galán and Ferrell 2013; Šegvić et al. 2018). Consequently, in the context of the URV, muscovite-based clay may be procured from the fluvioglacial, glaciolacustrine, colluvial, and till sediment abundantly available on the collinear and mountain belts, whereas illite forms through chemical weathering (Chamley 1989; Bergaya and Lagaly 2006; Galán and Ferrell 2013) and, as such, is expected to be found within URV’s horizons of pedogenized loess and/or in the alluvium of the Rhône and its tributaries (Burri 1955; Reynard et al. 2009; Viers et al. 2014; Stalder 2015; Stutenbecker et al. 2018). Mica-vermiculite that occurs in certain ceramic pastes (Table 5, Figs. 7 and 8) might have originated from prolonged weathering of Fe-mica (Novikoff et al. 1972; Fordham 1990). Vermiculitized mica in the matrix of PC32 might have originated from the weathering of rocks enriched in Fe-rich phases such as biotite and amphibole (Abreu and Vairinho 1990; Velde 1992; Arocena et al. 2012; Carloni et al. 2021). This kind of parent material may correlate with intrusive and metamorphic bodies of the Aar External Crystalline Massif and Penninic realm (Fig. 5; “Geological background” section) (Sartori et al. 2006; Hettmann et al. 2009; von Tscharner et al. 2016).

REE abundances broadly correspond to the types of clays used in pottery manufacturing (“Elemental composition” section). In general, clay minerals are known to have high concentrations of total REE (McLennan 1989) with muscovite-like minerals displaying a comparable higher retention potential of REE due to their layer charge (Andersson et al. 2004; Meunier 2005; Honty et al. 2008; Degryse and Braekmans 2014; Šegvić et al. 2021). In addition, the parent material also played a role in the total trace element content. This can be inferred based on U/Th ratio, which remains relatively unchanged during weathering and reflects the chemistry of the source (McLennan et al. 1993; Condie et al. 1995; Degryse and Braekmans 2014; Gaillardet et al. 2014). Based on U/Th ratios of analyzed ceramics, at least two types of source may therefore be envisaged: one enriched in U used to make the ceramics of Naters *‘Altersheim’* and another documented for the rest of the dataset (Fig. 10d). A diverse Th content in pottery samples define intra-group variability of this second type of parent material.

The two samples of daub from Sion *‘Petit-Chasseur III’* (PC85 and PC86) are compositionally different compared to the rest of sample set (“Results” section). Acquired data revealed the use of an illite-based carbonate-rich clay for building purposes. The unimodal grain size distribution testifies to the allochems that represent natural inclusions of the original clay. Furthermore, the selected raw material received little or no manipulation before it was utilized. The use of carbonate-rich clays for wall plastering has been reported from various Neolithic sites in NW Switzerland (di Pierro 2002; Maggetti 2009).

### Paste preparation recipes

This section presents the four paste preparation recipes (R) that were reconstructed for the analyzed pottery based on compositional data (R1-R4; Table 6). The type of clay and temper material were inferred from the discussion of the result of petrographic, mineralogical, and geochemical characterizations in the light of URV geology (“Discussion” section).

**Table 6:** Summary of the reconstructed paste preparation recipes (R)

R	Clay type	Temper type	Site	Temper material source	Temper manipulation
R1a	Illite or muscovite	Coarse sediment rich in granite particles	Petit-Chasseur necropolis *Samples PC11, PC12, PC20, PC23, PC25, PC34, PC35, PC38, PC47, PC49, PC52, PC69, PC70 (EBA II, III, IV)*	Till sediment (Rhône Glacier)	Lithological sorting by hand (removal of carbonates and selection of granite), fragmentation
	Illite or muscovite	Coarse sediment rich in granite particles	Sion ‘Sous-le-Scex’ *Samples SS01, SS02, SS03 (EBA IV)*	Till sediment (Rhône Glacier)	Lithological sorting by hand (removal of carbonates and selection of granite), fragmentation
	Illite	Coarse sediment rich in granite particles	Salgesch ‘Mörderstein’ *Samples SM04, SM08 (EBA II, III-IV)*	Till sediment (Rhône Glacier)	Lithological sorting by hand (removal of carbonates and selection of granite), fragmentation
R1b	Muscovite	Coarse sediment rich in fine-grained granite and granite particles	Petit-Chasseur necropolis *Sample PC21 (EBA II)*	Till sediment (Rhône Glacier)	Lithological sorting by hand (removal of carbonates and selection of granite and fine-grained granite), fragmentation
	Muscovite	Coarse sediment rich in fine-grained granite and granite particles	Sion ‘Petit-Chasseur III’ *Samples PC82, PC88, PC91, PC92, PC98, PC99* (*EBA II?, III-IV)*	Till sediment (Rhône Glacier)	Lithological sorting by hand (removal of carbonates and selection of granite and fine-grained granite), fragmentation
	Muscovite	Coarse sediment rich in fine-grained granite and granite particles	Sion ‘Sous-le-Scex’ *Sample SS04 (EBA IV)*	Till sediment (Rhône Glacier)	Lithological sorting by hand (removal of carbonates and selection of granite and fine-grained granite), fragmentation
	Muscovite	Coarse sediment rich in fine-grained granite and granite particles	Salgesch ‘Mörderstein’ *Samples SM05, SM06 (EBA)*	Till sediment (Rhône Glacier)	Lithological sorting by hand (removal of carbonates and selection of granite and fine-grained granite), fragmentation
	Muscovite	Coarse sediment rich in fine-grained granite and granite particles	Naters ‘Altersheim’ *Samples NA01, NA02, NA03, NA04, NA05, NA06, NA07, NA08, NA09, NA10 (EBA)*	Till sediment (Rhône Glacier)	Lithological sorting by hand (removal of carbonates and selection of granite and fine-grained granite), fragmentation
	Illite	Coarse sediment rich in fine-grained granite and granite particles	Vex ‘Le Château’ *Sample VX01 (EBA IV)*	Till sediment (Rhône Glacier)	Lithological sorting by hand (removal of carbonates and selection of granite and fine-grained granite), fragmentation
R1c	Illite	Coarse sediment rich in weathered granite	Petit-Chasseur necropolis *Samples PC56, PC57 (EBA III)*	Till sediment (Rhône Glacier)	Lithological sorting by hand (removal of carbonates and selection of weathered granite)
R1d	Illite or muscovite	Coarse sediment rich in quartz-feldspar gneiss and granite	Petit-Chasseur necropolis *Samples PC39, PC40, PC48, PC54, PC59, PC62, PC63, PC66 (EBA II, III, IV)*	Till sediment (Rhône Glacier)	Lithological sorting by hand (removal of carbonates and selection of quartz-feldspar gneiss and granite)
	Illite or muscovite	Coarse sediment rich in quartz-feldspar gneiss and granite	Sion ‘Petit-Chasseur III’ *Samples PC81, PC83, PC84, PC87, PC89, PC90, PC94, PC95* (*EBA II?, III-IV)*	Till sediment (Rhône Glacier)	Lithological sorting by hand (removal of carbonates and selection of quartz-feldspar gneiss and granite)
R1e	Vermiculitized mica and possibly illite	Coarse sediment rich in granite and amphibole gneiss particles	Petit-Chasseur necropolis *Samples PC32 and PC16, PC17, PC30, PC31, PC33 (EBA, EBA IV)*	Till sediment (Rhône Glacier)	Lithological sorting by hand (removal of carbonates and selection of amphibole gneiss and granite)
R2	Illite	Addition of coarse sediment rich in granite and limestone	Sion ‘Petit-Chasseur III’ *Samples PC96, PC97, PC100* (*EBA II?, III-IV)*	Till sediment (Rhône Glacier)	Fragmentation?
R3	Illite	Addition of coarse sediment rich in amphibolite particles	Rarogne ‘Heidnischbühl II’ *Samples RH01, RH02, RH03, RH04-07 (EBA II-IV)*	Till sediment (Rhône Glacier)	Lithological sorting by hand (removal of carbonates and selection of amphibolite)
R4	Illite, carbonate-rich	/	Sion ‘Petit-Chasseur III’ *Samples PC85, PC86 (EBA III-IV)*	/	/

The paste preparation recipe R1 (Table 6) was followed for manufacturing the pottery of fabric 1 var. 1 and 2, fabrics 2 to 4, and fabric 5 var. 1 and 2 (Table 3, “Ceramic petrography” section). It involved the tempering of raw clay—illite- or muscovite-based—with coarse and unsorted particles of intrusive and metamorphic rocks. Temper material was procured from Rhône Glacier’s deposits largely available in the URV, whose original composition was modified by carbonate removal. Likely, during the preparation of ceramic paste for pottery of fabric 1 var. 1 and 2 and fabric 2, the selected temper material was further fragmented to reduce the size of coarser particles. Based on the lithology of aplastic inclusions, it is possible to identify five recipe variants as follows (Table 6): (R1a) granite particles (samples of fabric 1 var. 1 and 2); (R1b) fine-grained granite (fabric 2); (R1c) weathered granite (fabric 3); (R1d) quartz-feldspar gneiss (fabric 4), and (R1e) amphibole gneiss (fabric 5 var. 1 and 2). In general, R1 was recognized in EBA II, III, and IV jars from the Petit-Chasseur necropolis and pottery from all settlement sites except for Rarogne *‘Heidnischbühl II’* (Table 6). This recipe is therefore widely diffused and suggests the EBA communities of the URV shared a common technical tradition.

The second paste preparation recipe, R2, is somewhat similar to R1 as potters tempered illitic clay with till sediment from Rhône Glacier’s deposits, possibly previously fragmented as well (Table 6). In this case however, carbonates were not removed but introduced in the ceramic paste. R2 was followed to make the pottery of fabric 1 var. 3 (PC96, PC97, and PC100; Table 3) and is solely documented in the ceramic assemblage of Sion *‘Petit-Chasseur III’*.

The pottery made according to R3 were fabricated by mixing an illitic clay with coarse and unsorted amphibolite particles procured from Rhône Glacier’s deposits (Table 6), which were depurated of its carbonate component. Ceramics display the paste characteristics of fabric 5 var. 3 (Table 3, “Fabric 5: amphibole-rich rocks” section) and all belong to the assemblage of Rarogne *‘Heidnischbühl II’*.

Lastly, R4 has been used to prepare wall plaster at Sion *‘Petit-Chasseur III’* (samples PC85 and PC86, fabric 6; Table 3). The original raw material was illite- and carbonate-rich and was not further manipulated.

**Fig. 11:**
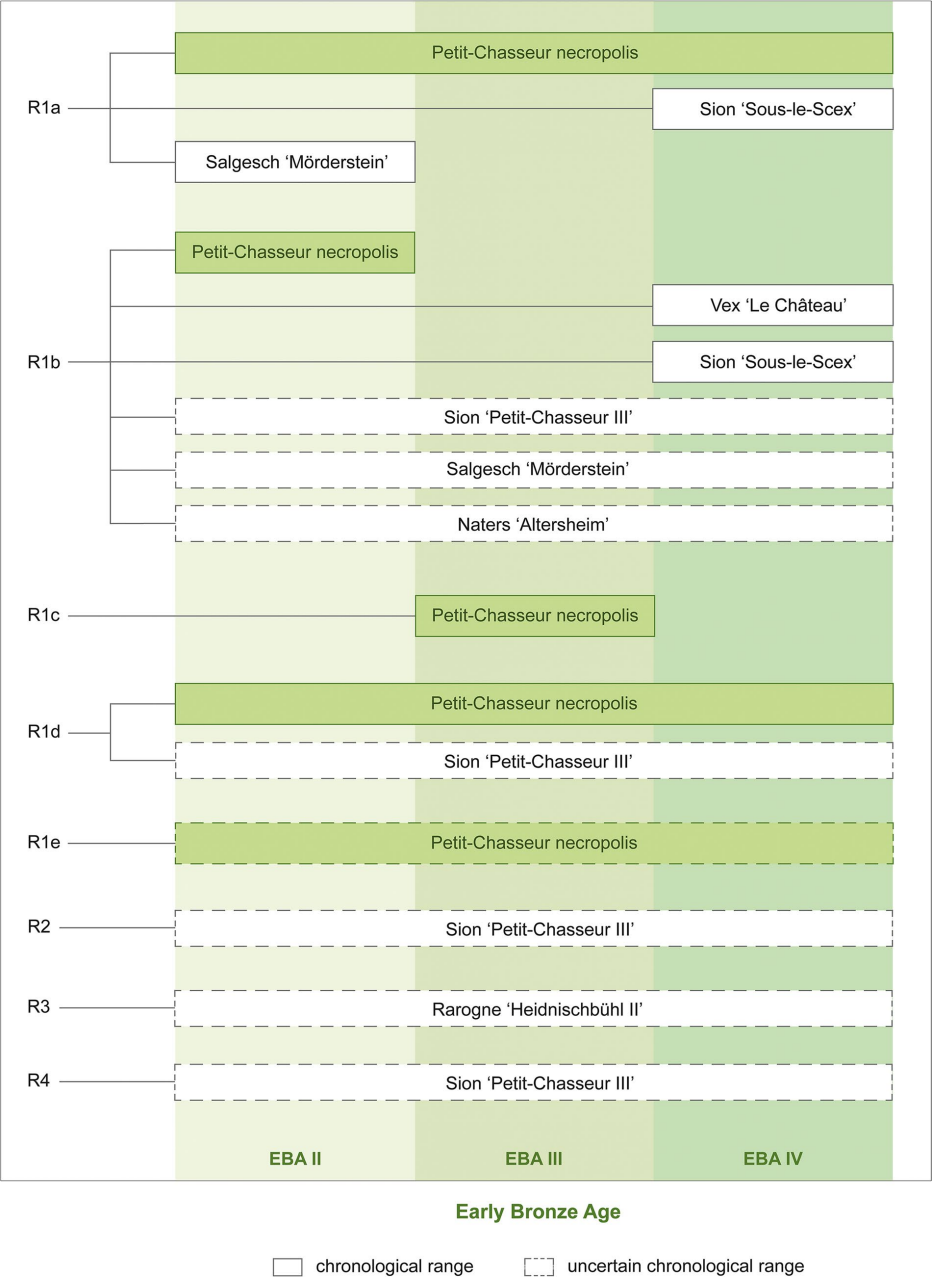
Chronology of the paste preparation recipes (R) listed in Table 6

**Fig. 12:**
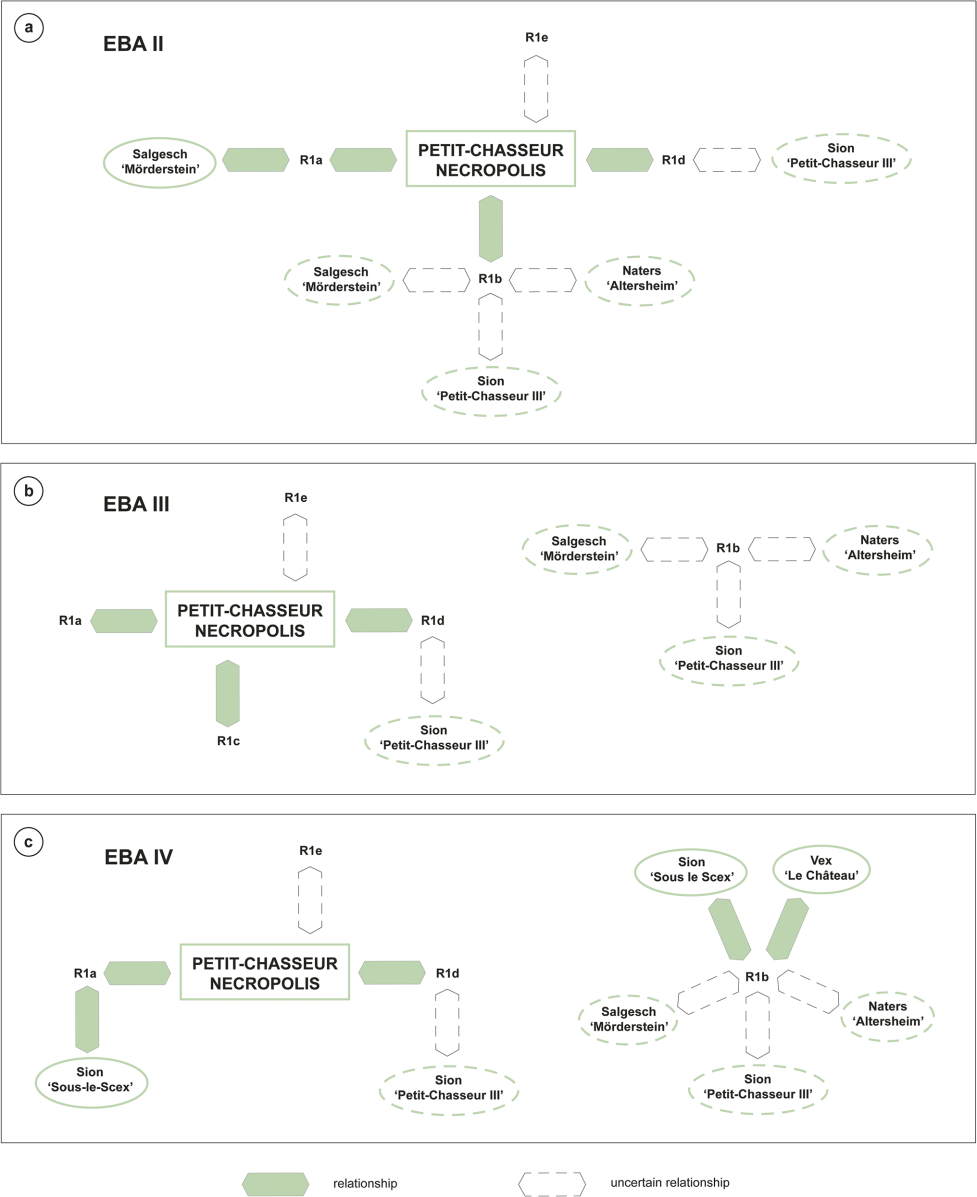
Network diagrams for the pottery from Petit-Chasseur necropolis and settlement sites built based on paste preparation recipes followed to make the ceramics: **a** phase EBA II; **b** phase EBA III; **c** phase EBA IV

Based on the chronology of analyzed pottery (Supplementary material 1; Tables 2 and 6), it is possible to discuss the use of the four paste preparation recipes throughout time (Fig. 11). R1 was used to make the jar offerings of the Petit-Chasseur over a long period that cover phases EBA II, III, and IV. Regarding the settlement sites, the doubts in phase attribution already expressed in “The settlement sites” sections for certain ceramic assemblages hamper the observation of the duration of these traditions in domestic contexts. For instance, the relative chronology of layers 4e and 4d of Sion *‘Petit-Chasseur III’* is still not fully understood. If an EBA III-IV occupation is certainly confirmed by ceramic typology, evidence of a phase EBA II is not conclusive (“The settlement sites” section). Uncertain chronological ranges also feature the analyzed body fragments from Naters *‘Altersheim’* (Supplementary material 1). Concerning R2, R3, and R4, their time frames are also undetermined based on archaeological data (Fig. 11). The network of relationships by phase is also uncertain given the doubt in phase attribution (Fig. 12). However, the diagrams obtained suggest that a plurality of relationships can be drawn between votive offerings and domestic pottery for each phase.

### Who venerated the ancestors? The ceramic viewpoint

Compositional data and reconstructed paste preparation recipes allowed one to compare the jar offerings from the Petit-Chasseur necropolis with the domestic pottery found at settlement sites (Table 6; Fig. 11). This research revealed that pottery used for daily and cultic activities was fabricated using similar raw materials, exploited and manipulated in comparable ways (“Results”, “Raw material choices”, and “Paste preparation recipes” sections). Paste preparation recipe R1 outlines a common technical tradition, shared between the communities of Sion *‘Petit-Chasseur III’*, Sion *‘Sous-le-Scex’*, Vex *‘Le Château’*, Salgesch *‘Mörderstein’*, and Naters *‘Altersheim’*. As outlined in “The settlement sites” section, the ceramic assemblages of these domestic-like contexts feature similar stylistic traits. Pottery typology and composition thus point to the existence of a common ceramic tradition shared at the regional scale. Choice of clay might have been influenced by available natural resources in the URV, but was only partially environment-dependent as calcareous clays were not used for pottery production (“Raw material choices” section). This does not, however, hold true for temper material. EBA potters selected one specific source of temper material—i.e. the morainic deposits left by the Rhône Glacier (Sect. 6.2.)—whereas they had at their disposal various sedimentary and metamorphic rocks outcropping in the vicinity of the sites (Fig. 5), and heterogeneous sandy sediments from fluvial deposits of the Rhône River and its tributaries (“Geological background” section). The selection of the till sediment from the Rhône Glacier’s deposits against the broad spectrum of resources locally available is, therefore, not uniquely environment-driven. The reasons are likely of a technological and cultural nature. In particular, the use of till sediment from Rhône Glacier’s deposits is also documented for the BB pottery deposited as grave goods in the megalithic monuments of Petit-Chasseur (Carloni et al. 2021). Hence, this work demonstrates that the EBA pottery was produced with an inherited way of doing, at least regarding ceramic raw material choice and use, which in turns testifies to the existence of a cultural filiation between the BB and EBA communities who had lived in the URV (Shennan 2000, 2013; Roux and Courty 2013; Roux 2019). The use of the rough-out technique (Derenne et al. 2020, 2022) and typology (cordoned jars, Besse 2003; Carloni et al. 2020) further bear testament to the transmission of specific traits of the pottery production. A cultural filiation between the BB and EBA societies in Europe is also supported by literature on the 3rd–2nd millennium BC historical scenario (Salanova et al. 2008; Kristiansen 2011, 2013; Fokkens and Harding 2013; Heyd 2013; Besse and Giligny 2021; Soares Lopes and Gomes 2021).

Notwithstanding the long-lasting tradition of raw material choice and use patterns, some important social tensions gave rise to a “desecration” of the Bell Beaker funerary monuments. At the beginning of the EBA in particular, some bell-shaped beakers from MXI were displaced outside the dolmen and the original entrance of the dolmen was permanently sealed. Bearing in mind the bell-shaped beakers were prestige items (e.g., Harrison and Heyd 2007; Kleijne 2019), their removal is an act full of meaning. Even if the sacredness of the funerary area was rapidly restored with new inhumations in the course of the EBA I phase (MXI and MIX, see Table 1), the characteristics of the latter mark a great shift in the funerary practices. First, the bell-shaped beaker/cup no more accompanied the dead as grave good. Second, the new burials exclusively consisted of individual inhumations and regarded a precise demography: two children, a fetus, and an adult woman. Third, the woman was accompanied by at least two jars. It is difficult to find an explanation for these changes, but they are probably related to an ongoing social process that drew attention to personal identities during the EBA (David-Elbiali 2000; Harrison and Heyd 2007; Brück and Fontijn 2013; Žegarac et al. 2021), which culminated in the EBA IV and gave rise to the individual graves placed in the vicinity of MVI (Table 1) (e.g., Harrison and Heyd 2007).

From a synchronic point of view, a common way of doing is reported between the EBA communities that lived in the URV. They shared a common ceramic tradition at least with regards to the two first steps of the *chaîne opératoire*—e.g., raw material acquisition and use (Roux 2019). Uncertainties in sample/site phase attribution hampered the proper enquiry of whether all the sites were simultaneously providing jars for the veneration activities that they took place at different stages at the Petit-Chasseur necropolis (Figs. 11 and 12). Pottery analyses showed clear similarities between the big containers deposited in the megalithic monuments and the domestic pottery of Sion *‘Petit-Chasseur III’*. Some ceramics, however, are made according to paste preparation recipe variants that find no comparison in the assemblage of this domestic context (i.e. R1a, R1c, and R1e; Fig. 12). The others that are attested at both Sion *‘Petit-Chasseur III’* and Petit-Chasseur necropolis are also shared with other EBA groups in the URV (e.g., R1b; Fig. 12). Although tempting, a unique and exclusive relationship between the community living at Sion *‘Petit-Chasseur III’* and the cultic activities conducted around the megalithic tombs is not supported by data on pottery composition. The same is true for the other settlement sites: no domestic context has a unique and exclusive relationship with the necropolis. On the contrary, acquired data on pottery composition support *a plurality of relationships*. Consequently, the monuments of the Petit-Chasseur necropolis do not exclusively belong to one single EBA group dwelling at ~ 100 m eastward—site of Sion *‘Petit-Chasseur III’*—but probably to the entire EBA society populating the valley. Besides, the exploitation of the same natural resources is indicative of a shared knowledge of the raw materials available in the environment and bear witness to the social interactions and ties between communities (Arnold 1985, 2018; Albero 2017). The similar characteristics of pottery fabrication process and style point out that Sion *‘Petit-Chasseur III’*, Sion *‘Sous-le-Scex’*, Vex *‘Le Château’*, Salgesch *‘Mörderstein’*, and Naters *‘Altersheim’* were part of the same community of practice (Wenger 1998). The position of the EBA group of Rarogne *‘Heidnischbühl II’* is more problematic in this scenario. Paste preparation recipe R3, used to make the cordoned jars found at the site, is similar to R1—which has been largely used for the jars of the Petit-Chasseur necropolis—in regard to procurement of temper material, which in both cases involved the Rhône Glacier’s deposits (“Paste preparation recipes” section). However, amphibolite particles of R3 are clearly distinguishable from (biotite-rich) granite, quartz-feldspar gneiss, and amphibole gneiss inclusions of R1. This suggests that potters from Rarogne *‘Heidnischbühl II’* may have exerted a different choice in regard to selection of temper material—amphibolite vs. leucocratic particles—and that they were part of a different community of practice. It also has to be highlighted that paste preparation recipes R1c and R1e are not represented in the ceramic assemblages from settlement sites known at present (“Paste preparation recipes” section; Figs. 11 and 12).

In conclusion, archaeometric pottery analyses disclosed that the Petit-Chasseur necropolis and its tombs probably belonged to the entire EBA community of the URV. A community featured by social complexity and sharing a common ceramic tradition, likely inherited from their predecessors—the BB groups—whose *élite* members were buried in the megalithic monuments that were perturbed, violated, and then venerated by depositing jars and faunal remains and with the erection of cairn structures. Jars—and their contents—were possibly offered to the forebears to request supernatural help or to give legitimacy to their power and normalize the current social order (Parker Pearson 1993). Within their tombs, the ancestors continued to inhabit the world of the living and to play a major role in society (Parker Pearson and Ramilisonina 1998; Scarre and Insoll 2011), further reinforcing ties among the individuals and the groups that shared a common landscape (Insoll 2011, and references therein). Gallay (1995, 2006, 2016) and Harrison and Heyd (2007) highlighted how far the Petit-Chasseur necropolis, with its standing dolmen and cists, and its millennial history, acted as a meaningful place in the 3rd and 2nd millennia BC. Through the lens of archaeometric pottery analyses, this study supports the hypothesis formulated in previous research (Carloni et al. 2021) that sees the EBA people gathering at the megalithic cemetery to venerate the ancestors living in disparate areas of the URV.

## Conclusions

Pottery analyses disclosed the EBA pottery of the URV was made using the raw material largely available in the region. Ceramic paste was prepared by tempering illitic or muscovitic clays with unsorted coarse particles from the Rhône Glacier’s deposits. Potters privileged rock particles composed of light- and dark-colored silicates (granite, fine-grained granite, quartz-feldspar gneiss, amphibole gneiss) and generally avoided carbonates. A total of four paste preparation recipes were reconstructed and served as a base to establish a link between the jars deposited as votive offerings in/around the megalithic tombs of the Petit-Chasseur necropolis and the domestic pottery. This work demonstrated that ceramics intended to be used for daily and cultic activities were fabricated using the same kinds of raw materials, exploited and manipulated in similar ways. Pottery composition thus points to the existence of a common ceramic tradition shared at a regional level. Raw material choices were only partially environment-dependent and largely driven by technological and cultural factors. Exploitation of the Rhône Glacier’s deposits may have been inherited by the BB groups that were burying the important figures of their society in the megalithic tombs of Petit-Chasseur, monuments that later became the object of an ancestor cult developed by EBA communities. From a synchronic point of view, strong similarities in important aspects of pottery production—i.e., raw material choices, paste preparation recipe, style—shows that the majority of the EBA groups documented throughout the valley shared a common ceramic tradition that was also used to make the jar votive offerings abandoned in/around the megalithic monuments. Through the lens of archaeometric pottery analyses, this work therefore proved that the EBA people venerating the ancestors at the Petit-Chasseur site were living in disparate areas of the URV and gathered at the megalithic cemetery to perform rituals.

## Supplementary Information

Below is the link to the electronic supplementary material.

Supplementary file1 (PDF 318 KB)

Supplementary file2 (PDF 257 KB)

Supplementary file3 (PDF 650 KB)

## Data Availability

Research datasets may be found in the manuscript and in Supplementary materials 1, 2, and 3.

## Footnotes

1 DMS geographical coordinates of Petit-Chasseur archaeological site: sector I-II 46°13′49.00988" N, 7°20′48.71855" E; dolmen MXII 46°13′53.06266" N, 7°20′54.77823" E. These coordinates were obtained by converting the Swiss projection coordinates published by Baudais et al. (1989) into the global WGS84 coordinates (GPS) using the NAVREF converter provided by the Federal Office of Topography of Switzerland.
