# Supplementary material for: Who venerated the ancestors at the Petit-Chasseur site? Examining Early Bronze Age cultic activities around megalithic monuments through the archaeometric analyses of ceramic findings (Upper Rhône Valley, Switzerland, 2200–1600 BC)
Source: Archaeol Anthropol Sci. 2023 Apr 20;15(5):62. doi: 10.1007/s12520-023-01737-0 (PMC10119256; doi:10.1007/s12520-023-01737-0)
Supplement: Supplementary file 1 — Supplementary file1 (PDF 318 KB) [file 12520_2023_1737_MOESM1_ESM.pdf]

Who venerated the ancestors at the Petit-Chasseur site? Examining Early Bronze Age cultic activities around megalithic monuments through the archaeometric analyses of ceramic findings (Upper Rhône Valley – Switzerland, 2200-1600 BC)

Delia Carloni[1], Branimir Šegvić[2], Mario Sartori[3], Giovanni Zanoni[2], Marie Besse[1]
1University of Geneva, Laboratory of Prehistoric Archaeology and Anthropology, Department F.-A. Forel for Environmental and Aquatic Sciences, Geneva, Switzerland
2Texas Tech University, Department of Geosciences, Lubbock, Texas, USA
3University of Geneva, Department of Earth Sciences, Geneva, Switzerland
Corresponding author: delia.carloni@unige.ch

Supplementary Material 1 List of samples, features, typology, and applied methods

**Sample**	**Site**	**Chronology**	**Structure**	**Inventory Number**	**Publication Number**	**Stratigraphic Layer**	**External Surface Color**	**Internal Surface Color**	**Fracture Color**	**Shape**	**Rim**	**Base**	**Rim diameter**	**Base diameter**	**Decoration**	**Decoration Position**	**Prehension**	**Prehension Position**	**Analytical Techniques**
PC09 (Carloni et al. 2021)	Sion, Petit-Chasseur I	Early Bronze Age III	MV	PC1 MV 244	1633	4D (secondary position, originary po	10YR 4/1 dark gray	10YR 4/1 dark gray	10YR 4/1 dark gray										ICP-MS
PC10 (Carloni et al. 2021)	Sion, Petit-Chasseur I	Early Bronze Age III	MV	PC1 MV 2347	1625, 2347-2348	4C (secondary position, originary po	10YR 5/2 grayish brown	10YR 4/1 dark gray	10YR 5/2 grayish brown, 10YR 4/1 gray								lug	body	ICP-MS
PC11 (Carloni et al. 2021)	Sion, Petit-Chasseur I	Early Bronze Age IV	MVI	PC1 MVI 2163	333	4A4	10YR 5/4 yellowish brown	10YR 5/4 yellowish brown	10YR 5/4 yellowish brown						cordon	body			ICP-MS, OM, XRD
PC12 (Carloni et al. 2021)	Sion, Petit-Chasseur I	Early Bronze Age IV	MXI	PC1 MXI S'83.104	1153, 1154	4D	10YR 4/2 dark grayish brown	10YR 4/2 dark grayish brown	10YR 3/1 very dark gray			flat		17 cm					ICP-MS, OM
PC13 (Carloni et al. 2021)	Sion, Petit-Chasseur I	Early Bronze Age	MXI	PC1 10013, MXI S'82	1142	unknown	10YR 3/2 very dark grayish brown	10YR 3/1 very dark gray	10YR 3/1 very dark gray	closed?	rounded				cordon	on the rim			ICP-MS
PC14 (Carloni et al. 2021)	Sion, Petit-Chasseur I	Early Bronze Age III	MXI	PC1 MXI int 1269	657	5A1/4MAJ	10YR 3/1 very dark gray	10YR 3/1 very dark gray	10YR 3/1 very dark gray	closed?	rounded				cordon	neck			ICP-MS
PC15 (Carloni et al. 2021)	Sion, Petit-Chasseur I	Early Bronze Age IV	MXI	PC1 MXI S'83.127	1158, 1168	4D	10YR 5/2 grayish brown	10YR 5/2 grayish brown	10YR 5/2 grayish brown	jar	flat		25 cm		cordon	neck			ICP-MS
PC16 (Carloni et al. 2021)	Sion, Petit-Chasseur I	Early Bronze Age IV	MXI	PC1 MXI S'83.125	1157	4D	10YR 5/4 yellowish brown, 10YR 3/1 very dark gray	10YR 3/1 very dark gray	10YR 5/4 yellowish brown	jar	flat				cordon	shoulder			ICP-MS, OM
PC17 (Carloni et al. 2021)	Sion, Petit-Chasseur I	Early Bronze Age IV	MXI	PC1 MXI S'83.134	1159	4D	10YR 3/1 very dark gray	10YR 4/3 brown	10YR 4/3 brown	jar	rounded				cordon	shoulder			ICP-MS, OM
PC18 (Carloni et al. 2021)	Sion, Petit-Chasseur I	Early Bronze Age IV	MXI	PC1 MXI S'83.114	1155	4D	10YR 4/2 dark grayish brown	10YR 4/2 dark grayish brown	10YR 4/2 dark grayish brown								lug	body?	ICP-MS
PC19 (Carloni et al. 2021)	Sion, Petit-Chasseur I	Early Bronze Age IV	MXI	PC1 MXI S'84.188	1171	4D	10YR 4/3 brown	10YR 5/4 yellowish brown	10YR 5/4 yellowish brown	jar	rounded				cordon	neck			ICP-MS
PC20 (Carloni et al. 2021)	Sion, Petit-Chasseur I	Early Bronze Age IV	MXI	PC1 MXI int. Jarre 6	906-909	4DMAJ - Dépôt 6	10YR 6/3 pale brown, 10YR 3/2 very dark grayish brown	10YR 3/2 very dark grayish brown	10YR 3/2 very dark grayish brown	jar	flat				cordon	body, vertical, horizontal	strap handle	shoulder	ICP-MS, OM
PC21 (Carloni et al. 2021)	Sion, Petit-Chasseur I	Early Bronze Age II	MXI	PC1 MXI int. Jarre 23	1020-1024	5A51MAJ - Dépôt 2	10YR 6/3 pale brown, 10YR 4/3 brown	10YR 6/3 pale brown, 10YR 4/3 brown	10YR 4/1 dark gray	jar	rounded	flat			cordon	shoulder	no. 2 lugs	on the cordon	ICP-MS, OM
PC22 (Carloni et al. 2021)	Sion, Petit-Chasseur I	Early Bronze Age IV	MXI	PC1 MXI S'84.106	1186	4D	7.5YR 5/3 brown	10YR 4/2 dark grayish brown	7.5YR 5/3 brown								lug	shoulder?	ICP-MS
PC23 (Carloni et al. 2021)	Sion, Petit-Chasseur I	Early Bronze Age IV	MXI	PC1 MXI int. Jarre 9	682-685	4DMAJ - Dépôt 6	10YR 6/3 pale brown	10YR 5/4 yellowish brown	10YR 5/4 yellowish brown	jar		flat			no. 4 cordons	body			ICP-MS, OM
PC24 (Carloni et al. 2021)	Sion, Petit-Chasseur I	Early Bronze Age III or IV	MVII	PC1 MVII 1977	1845	5A	10YR 6/3 pale brown	10YR 6/3 pale brown	GLEY1 4/N dark gray										ICP-MS
PC25 (Carloni et al. 2021)	Sion, Petit-Chasseur I	Early Bronze Age III or IV	MVII	PC1 MVII 1977	1843	4DMAJ	10YR 6/3 pale brown	10YR 6/3 pale brown	GLEY1 3/N very dark gray										ICP-MS, OM
PC26 (Carloni et al. 2021)	Sion, Petit-Chasseur I	Early Bronze Age IV	MXI	PC1 MXI V'82.65	1267	4D	GLEY1 4/N dark gray	10YR 3/1 very dark gray	GLEY1 3/N very dark gray						finger-impressed cordon?	body			ICP-MS
PC27 (Carloni et al. 2021)	Sion, Petit-Chasseur I	Early Bronze Age	MXI	PC1 10014, MXI S'82	1141	unknown	10YR 5/4 yellowish brown	10YR 4/2 dark grayish brown	10YR 3/1 very dark gray						cordon	body			ICP-MS
PC28 (Carloni et al. 2021)	Sion, Petit-Chasseur I	Early Bronze Age IV	MXI	PC1 MXI S'83.41	1149	4D	10YR 6/3 pale brown	10YR 6/3 pale brown	10YR 3/1 very dark gray	jar					cordon	body			ICP-MS
PC29 (Carloni et al. 2021)	Sion, Petit-Chasseur I	Early Bronze Age IV	MXI	PC1 MXI S'83.43	1150	4D	10YR 5/4 yellowish brown	10YR 5/4 yellowish brown	10YR 3/1 very dark gray						cordon	body	lug	on the cordon	ICP-MS
PC30 (Carloni et al. 2021)	Sion, Petit-Chasseur I	Early Bronze Age IV	MVI	PC1 MVI 2135	does not exist	4A2	10YR 3/3 dark brown	10YR 3/3 dark brown	10YR 3/3 dark brown										ICP-MS, OM
PC31 (Carloni et al. 2021)	Sion, Petit-Chasseur I	Early Bronze Age IV	MVI	PC1 MVI 2139	does not exist	4A	10YR 3/3 dark brown	10YR 3/3 dark brown	10YR 3/3 dark brown										ICP-MS, OM
PC32 (Carloni et al. 2021)	Sion, Petit-Chasseur I	Early Bronze Age	MVI	PC1 MVI 2140	does not exist	unknown	10YR 3/3 dark brown	10YR 3/3 dark brown	10YR 3/3 dark brown										ICP-MS, OM, SEM-EDS
PC33 (Carloni et al. 2021)	Sion, Petit-Chasseur I	Early Bronze Age	MVI	PC1 MVI 548	does not exist	unknown	10YR 3/2 very dark grayish brown	10YR 3/1 very dark gray	10YR 3/2 very dark grayish brown										ICP-MS, OM
PC34 (Carloni et al. 2021)	Sion, Petit-Chasseur I	Early Bronze Age IV	MVI	PC1 MVI 2174	does not exist	4A3	10YR 4/3 brown	10YR 4/1 dark gray	7.5YR 5/6 strong brown										ICP-MS, OM
PC35 (Carloni et al. 2021)	Sion, Petit-Chasseur I	Early Bronze Age IV	MVI	PC1 MVI 557	does not exist	4A1	10YR 6/4 light yellowish brown	10YR 3/1 very dark gray	10YR 6/4 light yellowish brown, 10YR 3/1 very dark gray										ICP-MS, OM
PC36 (Carloni et al. 2021)	Sion, Petit-Chasseur I	Early Bronze Age IV	MVI	PC1 MVI 556	does not exist	4A1	10YR 4/3 brown	10YR 3/1 very dark gray	10YR 2/1 black										ICP-MS
PC37 (Carloni et al. 2021)	Sion, Petit-Chasseur I	Early Bronze Age III	MV	PC1 MV 2350-2353 Sherd 2350	does not exist	unknown	10YR 4/1 dark gray	10YR 4/1 dark gray	GLEY1 4/N dark gray										ICP-MS
PC38 (Carloni et al. 2021)	Sion, Petit-Chasseur I	Early Bronze Age III	MXI	PC1 MXI int 1047	646	5A1/4MAJ	10YR 3/1 very dark gray	10YR 5/1 gray	10YR 5/4 yellowish brown								lug	body	ICP-MS, OM, XRD
PC39 (Carloni et al. 2021)	Sion, Petit-Chasseur I	Early Bronze Age II	MXI	PC1 MXI int 1046	645	5A51MAJ	10YR 5/4 yellowish brown	10YR 3/1 very dark gray	10YR 3/1 very dark gray			flat		10 cm					ICP-MS, OM
PC40 (Carloni et al. 2021)	Sion, Petit-Chasseur I	Early Bronze Age IV	MXI	PC1 MXI int 683	626	4DMAJ	10YR 3/1 very dark gray, 10YR 5/2 grayish brown	10YR 3/1 very dark gray	10YR 3/1 very dark gray	jar	flat?				cordon	neck			ICP-MS, OM
PC41 (Carloni et al. 2021)	Sion, Petit-Chasseur I	Early Bronze Age IV	MXI	PC1 MXI int 500, 827	607, 1041	4DMAJ	10YR 4/3 brown	10YR 3/1 very dark gray	10YR 3/1 very dark gray	jar	flat				cordon	neck			ICP-MS
PC42 (Carloni et al. 2021)	Sion, Petit-Chasseur I	Early Bronze Age IV	MXI	PC1 MXI int 188	578	4DMAJ	10YR 6/4 light yellowish brown, 10YR 5/1 gray	not preserved	7.5YR 5/6 strong brown						cordon	unknown			ICP-MS
PC43 (Carloni et al. 2021)	Sion, Petit-Chasseur I	Early Bronze Age III	MXI	PC1 MXI int 1268	656	5A1/4MAJ	10YR 4/1 dark gray, 10YR 4/6 strong brown	10YR 4/2 dark grayish brown	GLEY1 3/N very dark gray								lug	unknown	ICP-MS
PC44 (Carloni et al. 2021)	Sion, Petit-Chasseur I	Early Bronze Age IV	MXI	PC1 MXI int 497	605, 606	4DMAJ	10YR 5/2 grayish brown	10YR 4/2 dark grayish brown, 10YR 3/1 very dark gray	10YR 3/1 very dark gray						cordon	body			ICP-MS
PC45 (Carloni et al. 2021)	Sion, Petit-Chasseur I	Early Bronze Age III	MXI	PC1 MXI int 928	638	5A1/4MAJ	10YR 4/2 dark grayish brown	10YR 3/1 very dark gray	10YR 3/1 very dark gray	jar	flat, rounded		28 cm		no. 2 cordons	neck, body?	lug	on the cordon located on the body?	ICP-MS
PC46 (Carloni et al. 2021)	Sion, Petit-Chasseur I	Early Bronze Age IV	MXI	PC1 MXI int 360	594	4DMAJ	7.5YR 5/6 strong brown, 10YR 6/3 pale brown	10YR 3/1 very dark gray	7.5YR 5/6 strong brown, 10YR 3/1 very dark gray						cordon	unknown			ICP-MS
PC47 (Carloni et al. 2021)	Sion, Petit-Chasseur I	Early Bronze Age IV	MXI	PC1 MXI int 133	568	4DMAJ	10YR 4/3 brown, 10YR 5/1 gray	10YR 3/1 very dark gray	10YR 3/1 very dark gray						cordon	shoulder?	lug	on the cordon	ICP-MS, OM
PC48 (Carloni et al. 2021)	Sion, Petit-Chasseur I	Early Bronze Age IV	MXI	PC1 MXI int. Jarre 21	886-904	4DMAJ - Dépôt 6	10YR 6/1 gray AGG COLOR JAR	10YR 23/1 black	10YR 23/1 black	jar	rounded	flat			no. 3 cordons	1 under the rim, 2 shoulder	lug, strap handle	on the cordons	ICP-MS, OM, XRD, SEM-EDS
PC49 (Carloni et al. 2021)	Sion, Petit-Chasseur I	Early Bronze Age II	MXI	PC1 MXI int. Jarre 18	1009-1012	5A51MAJ - Dépôt 3	7.5YR 6/4 light brown	10YR 3/1 very dark gray	10YR 3/1 very dark gray	jar	rounded				cordon	shoulder	no. 2 lugs	on the cordon	ICP-MS, OM, XRD
PC50 (Carloni et al. 2021)	Sion, Petit-Chasseur I	Early Bronze Age III	MV	PC1 MV 214	1629	4D (secondary position, originary po	2.5YR 6/1 gray	/	/								lug	unknown	ICP-MS
PC51 (Carloni et al. 2021)	Sion, Petit-Chasseur I	Early Bronze Age III	MV	PC1 MV 230	does not exist	unknown	7.5YR 6/4 light brown	7.5YR 5/2 brown, 10YR 4/1 dark gray	GLEY1 4/N dark gray										ICP-MS
PC52 (Carloni et al. 2021)	Sion, Petit-Chasseur I	Early Bronze Age III	MV	PC1 MV 234	does not exist	unknown	7.5YR 6/4 light brown	7.5YR 5/2 brown, 10YR 4/1 dark gray	GLEY1 4/N dark gray	jar?									ICP-MS, OM
PC53 (Carloni et al. 2021)	Sion, Petit-Chasseur I	Early Bronze Age III	MV	PC1 MV 206	does not exist	unknown	10YR 6/3 pale brown	10YR 5/2 grayish brown	GLEY1 4/N dark gray										ICP-MS
PC54 (Carloni et al. 2021)	Sion, Petit-Chasseur I	Early Bronze Age III	MV	PC1 MV 2344	does not exist	unknown	7.5YR 5/4 brown	10YR 4/2 dark grayish brown	10YR 2/1 black										ICP-MS, OM
PC55 (Carloni et al. 2021)	Sion, Petit-Chasseur I	Early Bronze Age III	MV	PC1 MV 2395	does not exist	unknown	10YR 6/2 light brownish gray	GLEY1 4/N dark gray	GLEY1 4/N dark gray										ICP-MS
PC56 (Carloni et al. 2021)	Sion, Petit-Chasseur I	Early Bronze Age III	MV	PC1 MV 302-306, 308 Sherd 302	does not exist	unknown	2.5YR 5/1 gray	GLEY1 4/N dark gray	GLEY1 4/N dark gray										ICP-MS, OM, XRD, SEM-EDS
PC57 (sherd 245) (Carloni et al. 2021)	Sion, Petit-Chasseur I	Early Bronze Age III	MV	PC1 MV 245, 269	does not exist	unknown	10YR 6/3 pale brown	GLEY1 3/N very dark gray	GLEY1 3/N very dark gray										ICP-MS, OM
PC58 (Carloni et al. 2021)	Sion, Petit-Chasseur I	Early Bronze Age III	MV	PC1 MV 2398	does not exist	unknown	10YR 4/2 dark grayish brown	10YR 3/1 very dark gray	GLEY1 4/N dark gray										ICP-MS
PC59 (Carloni et al. 2021)	Sion, Petit-Chasseur I	Early Bronze Age II-III?	MXI	PC1 MXI R'84.83	1111	5ASUP	7.5YR 4/3 brown	7.5YR 5/6 strong brown	GLEY1 3/N very dark gray						cordon	shoulder?			ICP-MS, OM, XRD
PC60 (Carloni et al. 2021)	Sion, Petit-Chasseur I	Early Bronze Age II-III?	MXI	PC1 MXI S'84.209	1194	5ASUP	10YR 5/3 brown	10YR 5/4 yellowish brown, 10YR 4/2 dark grayish brown	GLEY1 4/N dark gray						cordon	shoulder?			ICP-MS
PC61 (Carloni et al. 2021)	Sion, Petit-Chasseur I	Early Bronze Age II-III?	MXI	PC1 MXI S'84.148	1192	5ASUP	10YR 5/3 brown	10YR 4/1 dark gray	10YR 4/1 dark gray						cordon	unknown			ICP-MS
PC62 (Carloni et al. 2021)	Sion, Petit-Chasseur I	Early Bronze Age IV	MXI	PC1 MXI S'82.129	1140	4D	7.5YR 5/4 brown	10YR 5/4 yellowish brown	10YR 5/4 yellowish brown						cordon	body?	lug	on the cordon	ICP-MS, OM
PC63 (Carloni et al. 2021)	Sion, Petit-Chasseur I	Early Bronze Age IV	MXI	PC1 MXI S'85.47	1199, 1200	4D	10YR 2/1 black, 10YR 3/3 dark brown	10YR 3/3 dark brown	10YR 2/1 black, 10YR 3/3 dark brown						cordon	body			ICP-MS, OM, XRD, SEM-EDS
PC64 (Carloni et al. 2021)	Sion, Petit-Chasseur I	Early Bronze Age IV	MXI	PC1 MXI R'83.18	1097, 1098	4D	10YR 4/2 dark grayish brown	10YR 4/2 dark grayish brown	10YR 3/1 very dark gray						cordon	shoulder?	lug	on the cordon	ICP-MS
PC65 (Carloni et al. 2021)	Sion, Petit-Chasseur I	Early Bronze Age II-III?	MXI	PC1 MXI R'82.105	1093	5ASUP	10YR 5/2 grayish brown	10YR 3/2 very dark grayish brown	10YR 3/1 very dark gray			flat							ICP-MS
PC66 (Carloni et al. 2021)	Sion, Petit-Chasseur I	Early Bronze Age IV	MXI - southern ad	PC1 MXI C. Adv Jarre 2	1292-1295	4D	10YR 4/2 dark grayish brown	10YR 3/1 very dark gray, 10YR 2/1 black	10YR 4/1 dark gray	jar	rounded				no. 2 cordons	under the rim, shoulder	no. 4 lugs	on the cordon located on the shoulder	ICP-MS, OM, XRD, SEM-EDS
PC67 (Carloni et al. 2021)	Sion, Petit-Chasseur I	Early Bronze Age II-III?	MXI	PC1 MXI R'81.1	1081	5ASUP	10YR 6/3 pale brown	10YR 3/1 very dark gray	10YR 3/1 very dark gray						cordon	unknown	lug	on the cordon	ICP-MS
PC68 (Carloni et al. 2021)	Sion, Petit-Chasseur I	Early Bronze Age II-III?	MXI	PC1 MXI R'83.60	1105	5ASUP	7.5YR 7/4 pink	GLEY1 3/N very dark gray	GLEY1 3/N very dark gray								lug	unknown	ICP-MS
PC69 (Carloni et al. 2021)	Sion, Petit-Chasseur I	Early Bronze Age III	MXI	PC1 MXI int 1229	654	5A1/4MAJ	10YR 5/2 grayish brown, 10YR 3/1 very dark gray	10YR 3/1 very dark gray	10YR 3/1 very dark gray	jar	rounded				cordon	under the rim	lug	shoulder	ICP-MS, OM, XRD
PC70 (Carloni et al. 2021)	Sion, Petit-Chasseur I	Early Bronze Age IV	MXI	PC1 MXI int 298	593	4DMAJ	7.5YR 6/6 reddish yellow	not preserved	10YR 7/6 reddish yellow	jar	with digital impression on				no. 2 cordons (one of the two is decorated with digital	under the rim, shoulder?			ICP-MS, OM
PC81	Sion, Petit-Chasseur III	Early Bronze Age III-IV	/	PC3 1897 Vase 1	/	4d	10YR 4/1 dark gray, 10YR 5/2 grayish brown, 10YR 6/3 pale brown	10YR 5/1 gray, 10YR 3/1 very dark gray	10YR 3/1 very dark gray	jar	rounded	flat	20 cm		cordon no. 2?	neck, shoulder?	lug no.2 or 4?	on the cordon on the shoulder?	ICP-MS, OM, XRD, SEM-EDS
PC82	Sion, Petit-Chasseur III	Early Bronze Age II?, III-IV	/	PC3 1987 Vase 2	/	4e2	7.5YR 6/4 light brown, 10YR 6/3 pale brown, 2.5YR 6/6 light red	7.5YR 6/4 light brown, 5YR 5/4 reddish brown, 5YR 6/4 light reddish brown	GLEY1 3/N very dark gray	jar	rounded		24 cm		cordon	neck	lugs, no. 3?	body?	ICP-MS, OM, XRD
PC83	Sion, Petit-Chasseur III	Early Bronze Age II?, III-IV	/	PC3 Vase 4	/	4e2	10YR 6/2 light brownish gray, 10YR 5/1 gray, 10YR 6/1 gray	10YR 3/1 very dark gray, 10YR 4/3 brown	10YR 3/1 very dark gray	amphore?	flat		15 cm		cordon no.2; plastic ring	neck, shoulder; shoulder	strap handle	shoulder	ICP-MS, OM, XRD, SEM-EDS
PC84	Sion, Petit-Chasseur III	Early Bronze Age III-IV	/	PC3 Vase 3	/	4d	10YR 6/2 light brownish gray, 10YR 4/1 dark gray, 10YR 4/2 dark grayish brown	10YR 3/1-4/1 dark gray, 10YR 6/2 light brownish gray	10YR 5/3 brown	jar	rounded	flat	11 cm		pastille aplati	body			ICP-MS, OM, XRD, SEM-EDS
PC85	Sion, Petit-Chasseur III	Early Bronze Age III-IV	/	PC3 I14-87	/	4d	7.5YR 6/6 reddish yellow	7.5YR 6/6 reddish yellow	7.5YR 6/6 reddish yellow	daub									LA-ICP-MS, OM, XRD, SEM-EDS
PC86	Sion, Petit-Chasseur III	Early Bronze Age III-IV	/	PC3 I16-31	/	4d	5YR 6/ light reddish brown	5YR 6/ light reddish brown	5YR 6/ light reddish brown	daub									LA-ICP-MS, OM, XRD
PC87	Sion, Petit-Chasseur III	Early Bronze Age III-IV	/	PC3 D13-110	/	4d	10YR 6/3 pale brown	10YR 6/3 pale brown	10YR 3/2 very dark grayish brown										ICP-MS, OM, XRD
PC88	Sion, Petit-Chasseur III	Early Bronze Age III-IV	/	PC3 F16-24	/	4d	10YR 4/2 dark grayish brown	10YR 6/4 light yellowish brown	GLEY1 4/N dark gray										LA-ICP-MS, OM
PC89	Sion, Petit-Chasseur III	Early Bronze Age III-IV	/	PC3 I15-195+117	/	4d	10YR 5/2 grayish brown	10YR 3/1 very dark gray	10YR 3/1 very dark gray										ICP-MS, OM, XRD
PC90	Sion, Petit-Chasseur III	Early Bronze Age III-IV	/	PC3 I15-137+143	/	4d	10YR 6/3 pale brown	10YR 3/1 very dark gray	10YR 3/1 very dark gray										ICP-MS, OM, XRD
PC91	Sion, Petit-Chasseur III	Early Bronze Age III-IV	/	PC3 I12-16	/	4d	7.5YR 6/4 light brown	10YR 5/2 grayish brown	7.5YR 6/4 light brown										LA-ICP-MS, OM
PC92	Sion, Petit-Chasseur III	Early Bronze Age II?, III-IV	/	PC3 H16-156	/	4e1-2	7.5YR 6/2 pinkish gray	10YR 4/2 dark grayish brown	GLEY1 4/N dark gray										ICP-MS, OM, XRD
PC93	Sion, Petit-Chasseur III	Early Bronze Age II?, III-IV	/	PC3 D14-9	/	4e1-2	10YR 6/3 pale brown	/	5YR 6/6 reddish yellow, 10YR 6/3 pale brown										LA-ICP-MS
PC94	Sion, Petit-Chasseur III	Early Bronze Age II?, III-IV	/	PC3 G12-138	/	4e1-2	10YR 6/3 pale brown	10YR 6/2 light brownish gray	10YR 6/3 pale brown			flat		7 cm					ICP-MS, OM
PC95	Sion, Petit-Chasseur III	Early Bronze Age II?, III-IV	/	PC3 F15-35	/	4e1-2	10YR 4/2 dark grayish brown	10YR 3/1 very dark gray	10YR 3/1 very dark gray			flat							ICP-MS, OM
PC96	Sion, Petit-Chasseur III	Early Bronze Age II?, III-IV	/	PC3 H12-56+58	/	4e1-2	10YR 6/3 pale brown	10YR 6/3 pale brown, 10YR 4/1 dark gray	10YR 6/3 pale brown										ICP-MS, OM, XRD
PC97	Sion, Petit-Chasseur III	Early Bronze Age II?, III-IV	/	PC3 H12-58	/	4e1-2	10YR 6/3 pale brown	10YR 6/3 pale brown, 10YR 3/1 very dark gray	10YR 6/3 pale brown, 10YR 3/1 very dark gray										ICP-MS, OM, XRD
PC98	Sion, Petit-Chasseur III	Early Bronze Age II?, III-IV	/	PC3 G19-16	/	4e1-2	10YR 4/2 dark grayish brown?	10YR 4/2 dark grayish brown	10YR 2/1 black										LA-ICP-MS, OM
PC99	Sion, Petit-Chasseur III	Early Bronze Age II?, III-IV	/	PC3 I15-58	/	4e1-2	10YR 3/1 very dark gray	10YR 3/2 very dark grayish brown	10YR 3/1 very dark gray										ICP-MS, OM, XRD
PC100	Sion, Petit-Chasseur III	Early Bronze Age II?, III-IV	/	PC3 H12-56+85	/	4e1-2	10YR 6/3 pale brown	10YR 6/3 pale brown, 10YR 4/1 dark gray	10YR 6/3 pale brown, 10YR 4/1 dark gray										ICP-MS, OM, XRD, SEM-EDS
NA01	Naters 'Altersheim'	Early Bronze Age	Pit UT7	NA04.34	/	UT7	10YR 5/4 yellowish brown	10YR 5/3 brown, 10YR 4/1 dark gray	GLEY1 4/N dark gray										ICP-MS, OM, XRD
NA02	Naters 'Altersheim'	Early Bronze Age	Pit UT7	NA04.35	/	UT7	10YR 5/3 brown	10YR 4/2 dark grayish brown	GLEY1 4/N dark gray										ICP-MS, OM, XRD
NA03	Naters 'Altersheim'	Early Bronze Age	Pit UT7	NA04.36	/	UT7	10YR 5/3 brown	10YR 4/1 dark gray	GLEY1 4/N dark gray										ICP-MS, OM, XRD
NA04	Naters 'Altersheim'	Early Bronze Age	Pit UT7	NA04.37	/	UT7	10YR 5/4 yellowish brown	10YR 4/1 dark gray	10YR 4/1 dark gray										ICP-MS, OM, XRD
NA05	Naters 'Altersheim'	Early Bronze Age	Pit UT7	NA04.38	/	UT7	10YR 5/4 yellowish brown	10YR 4/1 dark gray	GLEY1 4/N dark gray										ICP-MS, OM, XRD, SEM-EDS
NA06	Naters 'Altersheim'	Early Bronze Age	Pit UT7	NA04.39	/	UT7	10YR 5/4 yellowish brown	/	GLEY1 4/N dark gray										ICP-MS, OM, XRD, SEM-EDS
NA07	Naters 'Altersheim'	Early Bronze Age	Pit UT7	NA04.40	/	UT7	10YR 5/4 yellowish brown	/	GLEY1 4/N dark gray										OM
NA08	Naters 'Altersheim'	Early Bronze Age	Pit UT7	NA04.41	/	UT7	10YR 5/3 brown	10YR 5/3 brown	GLEY1 4/N dark gray										OM, XRD

NA09	Naters ‘Altersheim’	Early Bronze Age	Pit UT7	NA04.42	/	UT7	5YR 5/4 reddish brown	10YR 6/3 pale brown	GLEY1 4/N dark gray										OM
NA10	Naters ‘Altersheim’	Early Bronze Age	Pit UT7	NA04.43	/	UT7	10YR 6/3 pale brown	10YR 2/1 black	GLEY1 4/N dark gray										OM
RH01	Rarogne ‘Heidnischbühl II’	Early Bronze Age III-IV	P42	HB II 6753	/	4	7.5YR 6/6 reddish yellow	10YR 6/3 pale brown	10YR 6/3 pale brown		rounded				cordon	neck			LA-ICP-MS, OM, XRD
RH02	Rarogne ‘Heidnischbühl II’	Early Bronze Age IV	d (1961)	HB II 4824	/	4inf	10YR 5/2 grayish brown	10YR 6/3 pale brown	GLEY1 3/N very dark gray		flat				cordon	neck			ICP-MS, OM, XRD
RH03	Rarogne ‘Heidnischbühl II’	Early Bronze Age II-IV	n (1961)	HB II 5816	/	4inf	10YR 5/3 brown, 7.5YR 6/6 reddish yellow	10YR 2/1 black	GLEY1 3/N very dark gray						cordon	unknown			ICP-MS, OM, XRD
RH04	Rarogne ‘Heidnischbühl II’	Early Bronze Age IV	P39	HB II Jarre P39	/	P39	10YR 3/1 very dark gray, 10YR 4/2 dark grayish brown	10YR 2/1 black	10YR 2/1 black	body fragment attributed to jar P39					cordon	shoulder	lug no. 2?	on the cordon	ICP-MS, OM, XRD
RH05	Rarogne ‘Heidnischbühl II’	Early Bronze Age IV	P39	HB II Jarre P39	/	P39	10YR 3/1 very dark gray, 10YR 4/2 dark grayish brown	10YR 2/1 black	10YR 2/1 black	body fragment attributed to jar P39									ICP-MS, OM, XRD
RH06	Rarogne ‘Heidnischbühl II’	Early Bronze Age IV	P39	HB II Jarre P39	/	P39	10YR 3/1 very dark gray, 10YR 4/2 dark grayish brown	10YR 2/1 black	10YR 2/1 black	body fragment attributed to jar P39									ICP-MS, OM, XRD, SEM-EDS
RH07	Rarogne ‘Heidnischbühl II’	Early Bronze Age IV	P39	HB II Jarre P39	/	P39	10YR 3/1 very dark gray, 10YR 4/2 dark grayish brown	10YR 2/1 black	10YR 2/1 black	body fragment attributed to jar P39									ICP-MS, OM, XRD
SM04	Salgesch ‘Mörderstein’	Early Bronze Age II	/	FM Vase 12	Vase 12	FOY434/CO40/REMB433/RU34	5YR 5/6 yellowish red, 10YR 5/4 yellowish brown	10YR 3/1-4/1 dark gray, 10YR 5/3 brown	5YR 5/6 yellowish red, 10YR 4/1 dark gray	jar	flat	flat	18 cm	12,6?	cordon	shoulder	lug	on the cordon	ICP-MS, OM, XRD, SEM-EDS
SM05	Salgesch ‘Mörderstein’	Early Bronze Age	/	FM Vase 53	Vase 53	OCC438-RUS345	7.5YR 5/4 brown, 10YR 5/3 brown	10YR 5/3 brown	GLEY1 3/N very dark gray										ICP-MS, OM, XRD, SEM-EDS
SM06	Salgesch ‘Mörderstein’	Early Bronze Age	/	FM Vase 64	Vase 64	OCC438-RUS345-REMB341/OCC431	10YR 5/3 brown	10YR 5/3 brown	10YR 3/1 very dark gray										ICP-MS, OM, XRD
SM08	Salgesch ‘Mörderstein’	Early Bronze Age III-IV	/	FM Vase 26	Vase 26	CO40/REMB433	10YR 5/3 brown	10YR 5/3 brown	10YR 3/1 very dark gray						cordon				ICP-MS, OM, XRD
SS01	Sion ‘Sous-le-Scex’ "Sondage profond"	Early Bronze Age IV	/	SSS /D5 - SSS84 C3 6	/	10-déc.4	10YR 5/3 brown	10YR 4/2 dark grayish brown	GLEY1 4/N dark gray										LA-ICP-MS, OM, XRD
SS02	Sion ‘Sous-le-Scex’ "Sondage profond"	Early Bronze Age IV	/	SSS 84/D5 2	/	9-déc.4-5	10YR 4/1 dark gray, 10YR 6/3 pale brown	10YR 5/2 grayish brown	10YR 3/1 very dark gray										ICP-MS, OM, XRD, SEM-EDS
SS03	Sion ‘Sous-le-Scex’ "Sondage profond"	Early Bronze Age IV	/	SSS 84/D5 4	/	9-déc.4-5	7.5YR 5/4 brown	7.5YR 5/6 strong brown	10YR 3/1 very dark gray										LA-ICP-MS, OM
SS04	Sion ‘Sous-le-Scex’ "Sondage profond"	Early Bronze Age IV	/	SSS 84/D4 5	/	9-déc.4-5	10YR 5/4 yellowish brown	10YR 5/4 yellowish brown	10YR 4/1 dark gray										ICP-MS, OM, XRD, SEM-EDS
VX01	Vex ‘Le Château’	Early Bronze Age IV	/	VX 88 NO-10	/	Décapage 10	7.5YR 5/4 brown	10YR 5/2 grayish brown	GLEY1 4/N dark gray										LA-ICP-MS, OM, XRD, SEM-EDS

Notes

Chronology. The few doubts in Early Bronze Age phase attribution are due to stratigraphic or typological aspects.

Surface colors. The color codes were attributed by comparison with the Munsell Soil Color Charts 2009.
