# Supplementary material for: Who venerated the ancestors at the Petit-Chasseur site? Examining Early Bronze Age cultic activities around megalithic monuments through the archaeometric analyses of ceramic findings (Upper Rhône Valley, Switzerland, 2200–1600 BC)
Source: Archaeol Anthropol Sci. 2023 Apr 20;15(5):62. doi: 10.1007/s12520-023-01737-0 (PMC10119256; doi:10.1007/s12520-023-01737-0)
Supplement: Supplementary file 2 — Supplementary file2 (PDF 257 KB) [file 12520_2023_1737_MOESM2_ESM.pdf]

Who venerated the ancestors at the Petit-Chasseur site? Examining Early Bronze Age cultic activities around megalithic monuments through the archaeometric analyses of ceramic findings (Upper Rhône Valley – Switzerland, 2200-1600 BC)

Delia Carloni[1], Branimir Šegvić[2], Mario Sartori[3], Giovanni Zanoni[2], Marie Besse[1]

[1]University of Geneva, Laboratory of Prehistoric Archaeology and Anthropology, Department F.-A. Forel for Environmental and Aquatic Sciences, Geneva, Switzerland
[2]Texas Tech University, Department of Geosciences, Lubbock, Texas, USA
[3]University of Geneva, Department of Earth Sciences, Geneva, Switzerland
Corresponding author: delia.carloni@unige.ch

Supplementary Material 2 Petrographic fabric descriptions.

Granite (Samples PC11, PC12, PC20, PC23, PC25, PC34, PC35, PC38, PC47, PC49, PC52, PC69, PC70, PC96, PC97, PC100, SM04, SM08, SS01, SS02, SS03)

Variant 1 (Samples: PC11, PC12, PC20, PC23, PC25, PC34, PC35, PC38, PC47, PC69, PC70)

Inclusions

13-20%. Equant and elongate angular to rounded. Size <4 mm. Mostly single-spaced or less, double-spaced in samples PC20 and PC69. Not aligned to margins of samples. Trimodal in samples PC11, PC12, PC25, PC38, PC47, PC69 (mode 1 = 0.04 mm; mode 2 = 0.25 mm; mode 3 = 2.5 mm) and bimodal in samples PC20, PC23, PC34, PC35, PC70 (mode 1 = 0.04 mm; mode 2 = 0.3 mm), very poorly sorted grain size distribution.

Coarse fraction

4-0.06 mm

Dominant: Biotite-rich granite and granite; equant and elongate angular to subangular, <4 mm, mode = 2 mm. Very poorly sorted and homogeneously distributed.

Frequent: /

Common: Fine-grained granite with secondary calcite; equant and elongate subrounded to rounded, <3 mm, mode = 1.5 mm. Composed of quartz and feldspar accompanied by secondary calcite infillings. Moderately sorted and homogeneously distributed.

Few: Quartz; equant and elongate angular to subrounded, <0.9 mm. Poorly sorted and homogeneously distributed.

Very Few: /

Rare: Quartz schist; equant subrounded, <2 mm. Moderately sorted and inhomogenously distributed.

Mica schist; equant and elongate subangular to rounded, <0.8 mm. Moderately sorted and inhomogenously distributed.

Chlorite schist; equant and elongate subrounded to rounded, <1.5 mm. Poorly sorted and scatteredly distributed.

Feldspar; equant angular to subangular, <2 mm. Moderately sorted and scatteredly distributed.

Very Rare: Biotite; equant and elongate angular, <0.7 mm. Moderately sorted and inhomogeneously distributed.

Carbonate mudstone; equant subrounded, <2.5 mm.

Fe-rich carbonate mudstone; equant subrounded, <1.5 mm.

Sandstone; equant and elongate subrounded, <3 mm. It contains volcanic rock fragments.

Fine fraction

0.06-0.01 mm

Dominant:	Quartz
Frequent:	/
Common:	/
Few:	White mica; elongate angular. Sorted and homogeneously distributed.
Very Few:	/
Rare:	/
Very Rare:	/

Matrix

73-82%. Non-calcareous. Yellowish-brown or reddish-brown in PPL and XP. Heterogeneous. Optically slightly or moderately active.

Voids

3-7%. Mega and macro planar voids or channels, macro and meso vughs. Random orientation, aligned to margins in samples PC23, PC25, PC38, and PC69.

Variant 2 (Samples: PC49, PC52, SM04, SM08, SS01, SS02, SS03)

Inclusions

10-25%. Equant and elongate angular to rounded. Size <8 mm. Single-spaced. Not aligned to margins of samples. Trimodal in samples PC11, PC12, PC25, PC38, PC47, PC69 (mode 1 = 0.03 mm; mode 2 = 0.2 mm; mode 3 = 3 mm) and bimodal in samples PC20, PC23, PC34, PC35, PC70 (mode 1 = 0.03 mm; mode 2 = 0.3 mm), very poorly sorted grain size distribution.

<u>Coarse fraction</u>

8-0.06 mm

Dominant:	Biotite-rich granite; equant and elongate angular to rounded, <8 mm, mode = 3 mm. Very poorly sorted and homogeneously distributed.
Frequent:	/
Common:	/
Few:	Quartz; equant and elongate angular to subrounded, <1.5 mm. Poorly sorted and homogeneously distributed.
Very Few:	Feldspar; equant and elongate angular to subrounded, <2.5 mm. Very poorly sorted and scatteredly distributed.

Rare: Mica schist; elongate subangular, <6.5 mm. Moderately sorted and inhomogenously distributed.

Very Rare: Biotite; elongate angular to subangular, <1.5 mm. Moderately sorted and homogeneously distributed.

Cataclasite; equant subrounded, <6 mm. Moderately sorted and homogeneously distributed.

Fine fraction

0.06-0.01 mm

Dominant: Quartz

Frequent: /

Common: /

Few: White mica; elongate angular. Sorted and homogeneously distributed.

Very Few: /

Rare: /

Very Rare: /

Matrix

65-87%. Non-calcareous. Yellowish-brown to reddish-brown in PPL and XP. Heterogeneous. Optically slightly active.

Voids

3-10%. Mega, macro and meso channels, macro and meso vughs; mega, macro, and meso planar voids in sample PC52. Random orientation; aligned to margins in samples PC49, PC52, and SM08.

Variant 3 (Samples: PC96, PC97, PC100)

Inclusions

15-20%. Equant and elongate angular to rounded. Size <4.3 mm. Single-spaced. Not aligned to margins of samples. Polymodal (mode 1 = 0.04, mode 2 = 0.3 mm, mode 3 = 2.5 mm, mode 4 = 4.5 mm), very poorly sorted grain size distribution.

Coarse fraction

4.3-0.06 mm

Dominant: Granite; equant and elongate angular to rounded, <3.9 mm, modes = 2.5 and 4.5 mm. Very poorly sorted and homogeneously distributed.

Frequent: Micritic limestone; equant and elongate angular to rounded, <4.3 mm, modes = 0.25 and 2.5 mm. Very poorly sorted and homogeneously distributed.

Common: /

Few: Quartz; equant and elongate angular to subrounded, <0.8 mm. Poorly sorted and homogeneously distributed.

Very Few: Carbonate mudstone; equant and elongate rounded to subrounded, <1.5 mm. Poorly sorted and scatteredly distributed.

Rare: Mica schist; elongate angular, <1.4 mm. Moderately sorted and homogenously distributed.

Chlorite schist; equant and elongate subrounded to rounded, <0.7 mm. Moderately sorted and scatteredly distributed.

Very Rare: /

<u>Fine fraction</u>

0.06-0.01 mm

Dominant: Quartz

Frequent: /

Common: /

Few: White mica; elongate angular. Sorted and homogeneously distributed.

Very Few: /

Rare: /

Very Rare: /

Matrix

75-80%. Non-calcareous. Yellowish-brown in PPL and XP. Heterogeneous. Optically slightly active.

Voids

5%. Macro and meso channels and vughs. Random orientation.

Comments

This fabric is featured by presence of granitic rocks, whose particles' shape and degree of roundness point out they underwent a certain transportation. These are accompanied by other kinds of inclusions that correlate with other fabrics, in which they are present in different proportions and accompanied by other types of aplastic inclusions. Particles' morphometric characteristics, bimodal to polymodal grain-size distribution suggest the

tempering of the original raw material with an unsorted coarse sediment. The matrix is non-calcareous and yellowish- or reddish-brown in color. Firing conditions probably consisted into low to moderate temperatures and mostly reducing atmosphere. Preferential orientation of elongated voids may indicate the use of coils preformed by percussion on wet clay.

Fine-grained granite rich in Fe-oxide (Samples NA01, NA02, NA03, NA04, NA05, NA06, NA07, NA08, NA09, NA10, PC21, PC82, PC88, PC91, PC92, PC98, PC99, SM05, SM06, SS04, VX01)

Variant 1 (Samples: NA02, NA03, NA04, NA05, NA06, NA07, NA08, NA10, SS04)

Inclusions

10-20%. Equant and elongate angular to rounded. Size <5.8 mm (<1.3 mm in sample SS04). Single-spaced or double-spaced. Not aligned to margins of samples. Trimodal in samples NA03, NA05, NA06, NA07 (mode 1 = 0.04 mm; mode 2 = 0.2 mm; mode 3 = 2 mm) and bimodal in samples NA04, NA08, NA10, and SS04 (mode 1 = 0.04 mm; mode 2 = 0.2 mm), very poorly sorted grain size distribution.

Coarse fraction

5.8-0.06 mm (1.3-0.06 mm in sample SS04)

Dominant: Fine-grained granite with Fe-oxide; equant and elongate angular to rounded, <3.7 mm, mode = 2 mm. Composed of quartz and feldspar accompanied by Fe-oxide, particles show a cataclastic texture. Very poorly sorted and homogeneously distributed.

Frequent: /

Common: Granite; equant and elongate angular to subrounded, <4 mm. Composed of quartz and feldspar, slightly weathered. Very poorly sorted and homogeneously distributed.

Few: Quartz; equant and elongate angular, <0.3 mm. Moderately sorted and homogeneously distributed.

Very Few: Quartz and feldspar gneiss; equant and elongate angular to subrounded, <5.8 mm. Poorly sorted and scatteredly distributed.

Rare: Mica schist; elongate angular, <2.5 mm. Poorly sorted and homogenously distributed.

Very Rare: Biotite; elongate angular to subangular, <0.3 mm. Poorly sorted and homogeneously distributed.

Fine fraction

0.06-0.01 mm

Dominant: Quartz

Frequent: /

Common: /

Few: White mica; elongate angular. Sorted and homogeneously distributed.

Very Few: /

Rare: /

Very Rare: /

Matrix

77-87%. Non-calcareous. Dark yellowish-brown to dark brown in PPL and XP. Homogeneous. Optically slightly active.

Voids

3-5%. Mega and macro channels, macro and meso vughs. Random orientation.

Variant 2 (Samples: PC21, PC82, PC88, PC91, PC92, PC98, PC99, NA01, NA09, SM05, SM06, VX01)

Inclusions

10-25%. Equant and elongate angular to rounded. Size <6 mm. Single-spaced or double-spaced. Not aligned to margins of samples. Polymodal in sample PC99 (mode 1 = 0.03 mm; mode 2 = 0.2 mm; mode 3 = 4.5 mm), trimodal in samples PC21, PC82, PC88, PC92, VX01 (mode 1 = 0.03 mm; mode 2 = 0.2 mm; mode 3 = 2.5 mm) and bimodal in samples NA01, NA09, PC91, PC98, and SM05 (mode 1 = 0.03 mm; mode 2 = 0.2 mm), very poorly sorted grain size distribution.

Coarse fraction

6-0.06 mm

Dominant: Biotite-rich granite; equant and elongate angular to subangular, <5.2 mm, mode = 2.5 mm. Very poorly sorted and homogeneously distributed.

Frequent: Fine-grained granite with Fe-oxide; equant and elongate subrounded, <3.4 mm, mode = 2 mm. Composed of quartz and feldspar accompanied by Fe-oxide, particles show a cataclastic texture. Very poorly sorted and homogeneously distributed.

Common: /

Few: Quartz; equant and elongate angular to subrounded, <0.8 mm. Poorly sorted and homogeneously distributed.

Very Few: Feldspar; equant and elongate angular to subangular, <0.5 mm. Very poorly sorted and scatteredly distributed.

Rare: Mica schist; elongate angular, <0.6 mm. Moderately sorted and homogenously distributed.

Very Rare: Biotite; elongate angular to subangular, <2.2 mm. Moderately sorted and homogeneously distributed.

Phosphate; equant subangular to subrounded, <1.6 mm. Poorly sorted and homogeneously distributed.

Carbonate; equant and elongate subangular to subrounded, <1.1 mm. Moderately sorted and scatteredly distributed.

Fine fraction

0.06-0.01 mm

Dominant: Quartz

Frequent: /

Common: /

Few: White mica; elongate angular. Sorted and homogeneously distributed.

Very Few: /

Rare: /

Very Rare: /

Matrix

70-87%. Non-calcareous. Yellowish-brown to reddish brown in PPL and XP. Heterogeneous. Optically slightly active.

Voids

3-5%. Mega, macro and meso channels, macro and meso vughs. Random orientation; aligned to margins in samples NA01, PC21, PC82, PC88, and SM06.

Comments

This fabric is featured by presence of fine-grained granite rich in Fe-oxide, whose particles' shape and degree of roundness point out they underwent a certain transportation. These are accompanied by biotite-rich granite in the coarse fraction. Particles' morphometric characteristics, bimodal to trimodal grain-size distribution suggest the tempering of the original raw material with an unsorted coarse sediment. The color of the matrix and the degree of optical activity suggest the firing occurred in a mostly reducing atmosphere and at moderate firing temperatures. Preferential orientation of elongated voids may indicate the use of coils preformed by percussion on wet clay.

Weathered granite (Samples PC56, PC57)

Inclusions

20%. Equant and elongate shapes with angular to subrounded edges. Size <6.5 mm. Single-spaced or less. Not aligned to margins of samples. Trimodal (mode 1 = 0.05 mm; mode 2 = 0.3 mm; mode 3 = 2 mm), very poorly sorted grain size distribution.

Coarse fraction

6.5-0.06 mm

Dominant: Weathered granite; equant and elongate subangular to subrounded, <6.5 mm, mode = 2.5 mm. Composed of quartz, altered feldspar, and infillings of secondary calcite. Very poorly sorted and homogeneously distributed.

Frequent: /

Common: /

Few: Quartz; equant and elongate angular to subangular, < 0.1 mm. Poorly sorted and inhomogeneously distributed.

Very Few: Feldspar; equant angular to subangular, <1 mm. Poorly sorted and inhomogeneously distributed.

Rare: /

Very Rare: /

Fine fraction

0.06-0.01 mm

Dominant: Quartz; equant subrounded, <0.06 mm.

Frequent: /

Common: /

Few: White mica; elongate angular. Sorted and scatteredly distributed.

Very Few: /

Rare: /

Very Rare: /

Matrix

70-75%. Non-calcareous. Yellowish-brown to reddish brown in PPL and XP. Heterogeneous. Optically moderately active.

Voids

5-10%. Macro channels and meso vughs. Mostly random orientation, some channels aligned to margins in sample PC56.

Comments

This fabric is featured by presence of quartz and feldspar gneiss, whose particles' shape and degree of roundness point out they underwent a certain transportation. These are accompanied by granitic rocks in the coarse fraction. Particles' morphometric characteristics, trimodal grain-size distribution suggest the tempering of the original raw material with an unsorted coarse sediment. The color of the matrix and the degree of optical activity suggest the firing occurred in a mostly reducing atmosphere and at moderate firing temperatures. Preferential orientation of elongated voids may indicate the use of coils preformed by percussion on wet clay.

Quartz-feldspar gneiss (Samples PC39, PC40, PC48, PC54, PC59, PC62, PC63, PC66, PC81, PC83, PC84, PC87, PC89, PC90, PC94, PC95)

Variant 1 (Samples: PC89, PC90, PC94)

Inclusions

20-25%. Equant and elongate shapes with angular to rounded edges. Size <4.3 mm. Single-spaced or less. Not aligned to margins of samples. Trimodal in samples PC90 and PC94 (mode 1 = 0.06 mm; mode 2 = 0.2 mm; mode 3 = 1.5 mm) and bimodal in sample PC89 (mode 1 = 0.06 mm; mode 2 = 0.2 mm), very poorly sorted grain size distribution.

<u>Coarse fraction</u>

4.3-0.06 mm (3.3-0.06 mm in sample PC89)

Dominant: Quartz-feldspar gneiss; equant and elongate angular to rounded edges, <4.3-0.4 mm, mode = 2.5 mm (in sample PC89: <3.3 mm, mode = 1.5 mm). Composed of quartz, altered feldspar, and accessory phyllosilicate minerals such as white mica. Quartz crystals display undulose extinction. Very poorly sorted and homogeneously distributed.

Frequent: /

Common: Granite; equant subangular to rounded, 2.4-0.9 mm. Composed of quartz and feldspar, very weathered. Poorly sorted and homogeneously distributed.

Few: Quartz; equant angular to subrounded, straight extinction, < 0.6 mm. Moderately sorted and homogeneously distributed.

Very Few: /

Rare: /

Very Rare: Biotite; elongate subangular, 3.1 mm.

<u>Fine fraction</u>

0.06-0.01 mm

Dominant: Quartz; equant subrounded, straight extinction, < 0.06 mm.

Frequent: /

Common: /

Few: White mica; elongate angular. Sorted and scatteredly distributed.

Very Few: /

Rare: /

Very Rare: /

Matrix

70-77%. Non-calcareous. Yellowish-brown to dark brown in PPL, dark brown in XP. Heterogeneous. Optically moderately active.

Voids

3-5%. Macro and meso vughs and channels. Random orientation.

Variant 2 (Samples: PC40, PC48, PC66, PC81, PC83)

Inclusions

10-25%. Equant and elongate, angular to rounded. Size <5 mm. Mostly single-spaced, double-spaced in samples PC48, PC66, and PC83. Not aligned to margins of samples. Trimodal in samples PC40, PC66, and PC81 (mode 1 = 0.04 mm; mode 2 = 0.2 mm; mode 3 = 3mm) and bimodal in sample PC48 and PC83 (mode 1 = 0.04 mm; mode 2 = 0.2 mm), very poorly sorted grain size distribution.

<u>Coarse fraction</u>

5-0.06 mm (3-0.06 mm in samples PC48 and PC83)

Dominant: Quartz-feldspar gneiss; equant and elongate angular to rounded, <5 mm (in samples PC48 and PC83: <3 mm), mode = 2.5 mm. Composed of quartz, altered feldspar, and accessory phyllosilicate minerals such as white mica. Quartz crystals display undulose extinction. Very poorly sorted and homogeneously distributed.

Frequent: /

Common: Fine-grained granite with secondary calcite; equant subrounded to rounded, 0.8-0.25 mm (>5 mm in samples PC66), mode = 0.3 mm. Composed of quartz and feldspar accompanied by secondary calcite infillings. Moderately sorted and inhomogeneously distributed.

Few: Quartz; equant and elongate subangular to subrounded, <0.2 mm. Moderately sorted and homogeneously distributed.

Very Few: Mica schist; elongate angular to subangular, <1.8 mm. Moderately sorted and scatteredly distributed.

Rare: /

Very Rare: /

<u>Fine fraction</u>

0.06-0.01 mm

Dominant: Quartz; equant and elongate subangular to subrounded, <0.2 mm. Moderately sorted and homogeneously distributed.

Frequent: /

Common: /

Few: White mica; elongate angular, 0.03-0.02 mm. Sorted and homogeneously distributed.

Very Few: /

Rare: /

Very Rare: /

Matrix

68-82%. Yellowish brown or dark brown in PPL, dark brown in XP. Heterogeneous. Optically moderately active.

Voids

3-7%. Macro channels and macro and meso vughs. Random orientation, some channels aligned to margins in sample PC40.

Variant 3 (Samples: PC39, PC54, PC59, PC62, PC63, PC84, PC87, PC95)

Inclusions

10-20%. Equant and elongate, angular to rounded. Size <6.8 mm. Mostly double-space, single-spaced in samples PC54 and PC95. Not aligned to margins of samples. Trimodal in samples PC39, PC54, PC62, PC63, PC87 (mode 1 = 0.05 mm, mode 2 = 0.2 mm, mode 3 = 2 mm), bimodal in samples PC59, PC84, PC95 (mode 1 = 0.05 mm, mode 2 = 0.2 mm). Very poorly sorted grain size distribution.

<u>Coarse fraction</u>

6.8-0.06 mm (3-0.06 mm in sample PC59 and PC84)

Dominant: Biotite-rich granite or granite; equant and elongate angular to subrounded, <6.8 mm, mode = 2 mm. Composed of quartz and feldspar, and altered biotite, tectonized. Very poorly sorted and homogeneously distributed.

Frequent: Quartz-feldspar gneiss; equant and elongate angular to rounded, <4.3 mm, mode = 2.5 mm. Composed of quartz, altered feldspar, and accessory phyllosilicate minerals such as white mica. Quartz crystals display undulose extinction. Very poorly sorted and homogeneously distributed.

Common:

Few: Fine-grained granite with Fe-oxide; equant and elongate rounded to subrounded, 1.1-0.1 mm, mode = 0.5 mm. Composed of quartz and feldspar accompanied by Fe-oxide, particles show a cataclastic texture. Moderately sorted and homogeneously distributed.

Quartz; equant rounded to subrounded, <0.5 mm. Poorly sorted and homogeneously distributed.

Very Few: Biotite; elongate angular to subangular, 2.6-0.1 mm. Very poorly sorted and homogeneously distributed.

Rare: Mica schist; elongate angular to subangular, <0.5 mm. Moderately sorted and scatteredly distributed.

Feldspar; equant and elongate angular to subangular, <0.7 mm. Poorly sorted and inhomogeneously distributed.

Very Rare: Carbonate; equant rounded to subrounded, <0.1 mm. Sorted and scatteredly distributed.

<u>Fine fraction</u>

0.06-0.01 mm

Dominant: Quartz

Frequent: White mica; elongate angular, <0.06 mm. Sorted and homogeneously distributed.

Common: /

Few: /

Very Few: /

Rare: /

Very Rare:

Matrix

73-85%. Dark brown in PPL, dark grayish brown to black in XP. Heterogeneous. Optically slightly to moderately active.

Voids

3-7%. Macro channels and macro, meso, and micro vughs. Random orientation, some channels aligned to margins in all samples except for sample PC84.

Comments

This fabric is featured by presence of quartz and feldspar gneiss, whose particles' shape and degree of roundness point out they underwent a certain transportation. These are accompanied by granitic rocks in the

coarse fraction. Particles' morphometric characteristics, trimodal to bimodal grain-size distribution suggest the tempering of the original raw material with an unsorted coarse sediment. The color of the matrix and the degree of optical activity suggest the firing occurred in a mostly reducing atmosphere and at moderate firing temperatures. Preferential orientation of elongated voids may indicate the use of coils preformed by percussion on wet clay.

Amphibole-rich Rocks Fabric (Samples PC16, PC17, PC32, PC30, PC31, PC33, RH01, RH02, RH03, RH04, RH05, RH06, RH07)

Variant 1 (Samples: PC16, PC17, PC32)

Inclusions

15%. Equant and elongate, angular to subrounded. Size <5.4 mm (<3 mm in sample PC32). Single-spaced or less. Not aligned to margins of samples. Trimodal in samples PC16 and PC17 (mode 1 = 0.03 mm; mode 2 = 0.2 mm; mode 3 = 2 mm) and bimodal in sample PC32 (mode 1 = 0.03 mm; mode 2 = 0.2 mm), very poorly sorted grain size distribution.

Coarse fraction

5.4-0.07 mm (3-0.07 mm in sample PC32)

Dominant: Amphibole gneiss; equant and elongate angular and subangular, 5.4-0.5 mm, mode = 2 mm (in sample PC32: <2.3 mm, mode = 1.5 mm). Composed of albite and quartz, hornblende, and accessory minerals such as epidote and titanite. Quartz crystals display undulose extinction. Very poorly sorted and homogeneously distributed.

Frequent: /

Common: Quartz; two types:

- equant angular, undulose extinction, <1 mm, mode = 0.7 mm. Poorly sorted and homogeneously distributed.
- equant and elongate subangular, straight extinction, < 0.5 mm, mode = 0.3 mm. Moderately sorted and homogeneously distributed.

Few: Fine-grained granite with secondary calcite; equant subrounded, 1.3-0.25 mm, mode = 0.3 mm. Composed of quartz and feldspar accompanied by secondary calcite infillings. Poorly sorted and homogeneously distributed.

Very Few: Amphibole; elongate, subangular, <1.5 mm. It occurs in euhedral form, possibly identifiable as hornblende. Poorly sorting and inhomogeneous distribution in the samples.

Limestone; equant and elongate, subrounded, 2-0.5 mm. Poor sorting and inhomogeneous distribution in the samples.

Rare: /

Very Rare: Altered feldspar; equant and elongate angular and subangular, <1.5 mm. Moderately sorted and inhomogeneous distribution in the samples.

Biotite; elongate subangular, 1 mm.

Granite; elongate subrounded, 3-0.5 mm. Composed of quartz and feldspar, tectonized. Poorly sorted and scatteredly distributed.

Fine fraction

0.07-0.01 mm

Dominant: Quartz; equant and elongate subangular and subrounded, straight extinction, < 0.07 mm, mode = 0.03 mm. Moderately sorted and homogeneously distributed.

Frequent: /

Common: /

Few: /

Very Few: /

Rare: White mica; elongate angular, mode = 0.05 mm. Sorted and scatteredly distributed.

Very Rare: /

Matrix

78-82%. Non-calcareous. Brown to dark brown in PPL, brown to dark brown in XP. Homogeneous. Optically slightly active.

Voids

3-7%. Macro and meso planar voids and meso vughs. Mostly random orientation, some planar voids aligned to margins in sample PC17.

Variant 2 (Samples: PC30, PC31, PC33)

Inclusions

10-15%. Equant and elongate, angular to rounded. Size <3.2 mm. Mostly single-spaced. Not aligned to margins of samples. Trimodal in samples PC30 and PC33 (mode 1 = 0.06 mm; mode 2 = 0.2 mm; mode 3 = 2 mm) and bimodal in sample PC31 (mode 1 = 0.04 mm; mode 2 = 0.2 mm), very poorly sorted grain size distribution.

Coarse fraction

3.2-0.2 mm

Dominant: Biotite-rich granite; equant and elongate angular and subangular, 3-0.25 mm, mode = 2 mm (in sample PC32: <2.3 mm, mode = 1.5 mm). Composed of quartz and feldspar, and altered biotite, tectonized. Very poorly sorted and homogeneously distributed.

Frequent: /

Common: /

Few: Amphibole gneiss; equant and elongate subangular and subrounded, 2-1 mm, mode = 2 mm. Composed of albite and quartz, hornblende, and accessory minerals such as

epidote and titanite. Quartz crystals display undulose extinction. Poorly sorted and inhomogeneously distributed.

Very Few: Biotite; elongate angular, <1 mm. The mineral is slightly altered (vermiculitization).

Quartz; equant and elongate angular to subrounded, <0.5 mm. Very poorly sorted and homogeneously distributed.

Feldspar; equant and elongate angular and subangular, <0.5 mm. Poor sorting and scatter distribution.

Rare: Limestone; equant, subrounded and rounded, 0.5-0.2 mm. Moderate sorting and inhomogeneous distribution in the samples.

Mica schist; equant subrounded and rounded, 0.3-0.2 mm. Sorted and scatteredly distributed.

Very Rare: /

Fine fraction

0.2-0.01 mm

Dominant: Quartz; equant and elongate angular to subrounded. Moderately sorted and homogeneously distributed.

Frequent: /

Common: /

Few: /

Very Few: White mica; elongate angular, 0.05-0.02 mm. Sorted and scatteredly distributed.

Rare: Biotite

Very Rare: /

Matrix

75-87%. Brown to reddish brown in PPL, brown to reddish brown in XP. Heterogeneous. Optically moderately active.

Voids

3-7%. Macro and meso channels and meso vughs. Mostly random orientation, some channels aligned to margins in sample PC33.

Variant 3 (Samples: RH01, RH02, RH03, RH04, RH05, RH06, RH07)

Inclusions

20-30%. Equant and elongate, angular to subrounded. Size <4.9 mm. Single-spaced or less. Not aligned to margins of samples. Weakly polymodal in sample RH05 (mode 1 = 0.06 mm, mode 2 = 0.2 mm, mode 3 = 2

mm, mode 4 = 5 mm), trimodal in sample RH01, RH02, RH03, RH06, and RH07 (mode 1 = 0.05 mm, mode 2 = 0.2 mm, mode 3 = 3 mm), and bimodal in RH04 (mode 1 = 0.05 mm, mode 2 = 0.2 mm). Very poorly sorted grain size distribution.

Coarse fraction

4.9-0.05 mm (3.8-0.05 mm in sample RH04)

Dominant: Amphibolite; equant and elongate subangular to rounded, 4.9-0.25 mm, mode = 3 mm. Composed of hornblende, albite or alkali feldspar, ilmenite, and titanite. Very poorly sorted and homogeneously distributed.

Frequent: /

Common: Fine-grained granite with Fe-oxide; equant and elongate rounded to subrounded, 1.4-0.1 mm, mode = 0.5 mm. Composed of quartz and feldspar accompanied by Fe-oxide, particles show a cataclastic texture. Poorly sorted and homogeneously distributed.

Few: Granite; equant subangular, 2-0.2 mm. Composed of quartz and feldspar, tectonized. Poorly sorted and homogeneously distributed.

Very Few: Quartz; equant rounded to subrounded, <0.15 mm. Sorted and homogeneously distributed.

Rare: /

Very Rare: White mica, elongate angular, <0.2 mm. Sorted and homogeneously distributed.

Fine fraction

0.05-0.01 mm

Dominant: Quartz

Frequent: White mica

Common: /

Few: /

Very Few: /

Rare: /

Very Rare: /

Matrix

65-75%. Brown to very dark brown in PPL, dark grayish brown to black in XP. Heterogeneous. Optically slightly active.

Voids

5%. Mega, macro and meso vughs and macro and meso channels. Random orientation.

Comments

This fabric is featured by presence of high-grade metamorphic rocks of amphibolite facies. Particles' shape and degree of roundness point out they underwent a certain transportation. However, the very poor sorting suggests the distance was not long. Amphibolite-rich rocks are accompanied by granitic rocks. Particles' morphometric characteristics, bimodal to polymodal grain-size distribution suggest the tempering of the original raw material with an unsorted coarse sediment rich in amphibolite facies rocks and granite. The color of the matrix and the degree of optical activity suggest the firing occurred in a mostly reducing atmosphere and at moderate firing temperatures. Void arrangement do not allow for drawing inferences on the pots' forming techniques.

Allochem Fabric (Samples PC85, PC86)

Inclusions

5%. Equant and elongate, angular to rounded. Size <0.7 mm. Double-spaced. Not aligned to margins of samples. Unimodal, moderately sorted grain size distribution.

Dominant:	Allochem; equant and elongate rounded, subrounded, and subangular, mode = 0.2 mm. Inclusions composed of micritic calcite that lack a well-defined internal structure. Moderately sorted and homogeneously distributed in space.
Frequent:	Quartz; equant subangular and subrounded, mode = 0.04 mm. Inclusions shows internal fractures and fissures, moderate sorting, and homogeneous distribution.
Common:	/
Few:	/
Very Few:	White mica 3%; elongate angular, mode = 0.04 mm. Sorting and scatter distribution.
Rare:	Mica schist 2%; elongate subrounded, mode = 0.6 mm. Sorting and scatter distribution.
Very Rare:	/

Matrix

92-94%. Slightly calcareous. Brownish yellow to dark yellowish brown in PPL, dark yellowish brown in XP. Moderately homogeneous due to uneven distribution of micritic calcite. Optically slightly active.

Voids

1-3%. Macro channels and macro and meso vughs. Random orientation.

Comments

This fabric is carbonate-rich and characterized by presence of allochems in a slightly calcareous matrix. The unimodal grain size distribution of aplastic inclusions points out they represent natural inclusions of the original clay. Hence, the selected raw material received little or no manipulation before it was utilized. Slight matrix optical activity suggests the paste was subjected to firing.
